# Cognitive performance in offspring of parents with severe mental illness: a meta-analysis

**DOI:** 10.1017/S0033291726103985

**Published:** 2026-04-27

**Authors:** Akilew A. Adane, Abel Dadi, Alemken Jegnie, Abay W. Tadesse, Biruk Beletew Abate, Carrington C.J. Shepherd, Berihun Dachew

**Affiliations:** 1 Ngangk Yira Institute for Change, Murdoch University, Murdoch, Western Australia, Australia; 2 Menzies School of Health Research, Charles Darwin University, Casuarina, Northern Territory, Australia; 3 Addis Continental Institute of Public Health, Addis Ababa, Ethiopia; 4 The University of Western Australia, Crawley, Western Australia, Australia; 5 The Kids Research Institute Australia, Nedlands, Western Australia, Australia; 6 Curtin School of Population Health, Curtin University, Bentley, Western Australia, Australia; 7 Dream Science and Technology College, Dessie, Amhara region, Ethiopia; 8 Curtin Medical School, Curtin University, Bentley, Western Australia, Australia

**Keywords:** cognition, meta-analysis, offspring, parents, severe mental illness

## Abstract

Parental severe mental illnesses (SMIs), including schizophrenia, bipolar disorder, and major depressive disorder (MDD), can impact children’s well-being, yet existing meta-analyses are limited in scope and methodology and do not comprehensively assess cognitive and academic performance in offspring across SMIs. This meta-analysis aimed to synthesize the existing evidence on the association between parental SMIs and offspring cognitive and academic performance. MEDLINE, EMBASE, PsycINFO, and CINAHL were searched from their inception to December 2025. We included studies assessing associations between parental SMIs and offspring cognitive/academic performance at any age, including attention, memory, language, executive function, processing speed, IQ, social cognition, and academic performance. Standardized mean differences (SMDs) between offspring of parents with SMIs and controls were calculated. Differences in cognitive performance between affected offspring and controls were pooled using random-effects meta-analyses, with robust variance estimation. The meta-analysis included 109 studies (1,586,339 participants). Parental schizophrenia was strongly associated with several cognitive domains, including general cognition (SMD = −1.07, 95% CI: −1.92, −0.22), language (−0.70; −1.20, −0.20), and IQ (−0.53; −0.72, −0.34). Parental bipolar disorder was associated with general cognition (SMD = −0.45, 95% CI = −0.79, −0.12), memory (−0.40; −0.60, −0.19), executive function (−0.34; −0.51, −0.16), IQ (−0.32; −0.48, −0.17), and language (−0.18, 95% CI -0.34, −0.02). Parental MDD showed weaker but statistically significant associations with executive function, general cognition, and language development. Children of parents with SMIs, particularly schizophrenia or bipolar disorder, are at increased risk of cognitive difficulties. Population-level early intervention strategies targeting these families may improve offspring’s cognitive performance.

## Introduction

Severe mental illnesses (SMIs), including schizophrenia, bipolar disorder, and major depressive disorder (MDD), are debilitating chronic conditions that collectively affect over 247 million people globally (Global Burden of Disease Collaborators, 2022). The prevalence of these conditions places a significant burden on those affected and their families. Although individuals with SMIs are, on average, less likely to become parents than the general population, a considerable proportion do have children, resulting in a substantial number of offspring exposed to parental mental illness (Havelková, Havelka, & Koros Bartošová, 2024; Kravdal, Flatø, & Torvik, 2025; Stambaugh et al., 2017). Parental SMIs, particularly maternal mental illness, have been shown to negatively impact a range of perinatal outcomes with long-term consequences, including preterm birth, low birth weight, perinatal mortality, and congenital malformations (Adane et al., 2021.; Edvardsson et al., 2022; Etchecopar-Etchart, Mignon, Boyer, & Fond, 2022). Additionally, children of parents with SMIs are at an elevated risk of developing mental health issues, including behavioral disorders (Ayano, Betts, Maravilla, & Alati, 2021; Rasic, Hajek, Alda, & Uher, 2014; Uher et al., 2023; Zhou et al., 2024a).

Beyond perinatal complications and later mental health consequences, a growing body of evidence suggests that children of parents with SMIs, particularly those with schizophrenia, face challenges across multiple cognitive domains. These include deficits in executive function, processing speed, working memory, and general intelligence (Auerbach, Hans, & Zhang, 2022; Hemager et al., 2018; Knudsen et al., 2022; Lin et al., 2017a). These cognitive difficulties often persist into later childhood and adolescence, contributing to long-term developmental and educational challenges. The impact of parental SMIs on their offspring’s cognitive performance is thought to arise from a complex interplay of genetic and environmental factors (Jundong et al., 2012; Sandstrom, Sahiti, Pavlova, & Uher, 2019). Despite the increasing evidence, the literature remains inconsistent and fragmented, with many studies being small-scale and/or cross-sectional in design (De la Serna et al., 2020; Klimes-Dougan, Ronsaville, Wiggs, & Martinez, 2006; Restrepo-Mejia et al., 2023; Santucci et al., 2014, 2017).

Although there is a clear need for well-designed, adequately powered prospective longitudinal studies using representative population samples, synthesizing the existing evidence can offer more robust and generalizable conclusions about the cognitive and academic performance of children of parents with SMIs. Systematic reviews and meta-analyses play a key role in shaping public health strategies, informing prevention and intervention programs, and guiding policy development. However, despite the growing interest in this area, a comprehensive and methodologically rigorous meta-analytic synthesis remains lacking.

Existing reviews and meta-analyses are limited in several important ways, including being outdated, relying on narrative synthesis, focusing on narrow offspring age groups or specific parental conditions, and conflating distinct familial risk groups such as offspring, siblings, and other relatives (Agnew-Blais & Seidman, 2013; Bora et al., 2014; Bora & Özerdem, 2017; Hameed & Lewis, 2016; Klimes-Dougan, Jeong, Kennedy, & Allen, 2017; MacKenzie, Uher, & Pavlova, 2019; Pedruzo et al., 2025). Some early reviews are now dated, with literature searches ending more than a decade ago and conducted prior to substantial growth in population-based and longitudinal research, and were diagnostically narrow, focusing primarily on schizophrenia or psychosis (Agnew-Blais & Seidman, 2013; Bora et al., 2014). Several reviews were narrative in nature and did not undertake quantitative synthesis, reporting cognition as part of broader developmental or intellectual phenotypes without pooled, domain-specific effect estimates (Agnew-Blais & Seidman, 2013; Hameed & Lewis, 2016; Klimes-Dougan et al., 2017).

Meta-analyses that did quantify cognitive performance were often constrained by small numbers of studies and important methodological limitations, including reliance on specific offspring age groups without systematic evaluation across developmental stages, pooling of multiple correlated cognitive performances without robust adjustment for within-study non-independence, and inclusion of overlapping study populations (Bora et al., 2014; Bora & Özerdem, 2017; Pedruzo et al., 2025). In addition, some otherwise methodologically rigorous reviews were restricted to MDD rather than SMIs more broadly, limiting their relevance to offspring exposed to schizophrenia or bipolar disorder (MacKenzie et al., 2019). Notably, academic or educational performance has not been evaluated in offspring of parents with schizophrenia or bipolar disorder in existing meta-analyses.

Collectively, these limitations highlight the need for an updated synthesis that comprehensively evaluates cognitive domains, including language; incorporates academic and educational performance; focuses specifically on offspring across developmental stages; encompasses both psychotic and non-psychotic SMIs; and applies robust analytic approaches that appropriately address non-independence among outcomes. Such an approach would provide a more precise, developmentally informed, and methodologically rigorous assessment of cognitive and academic performance in children of parents with SMIs.

To address these gaps, we conducted a systematic review and meta-analysis to synthesize the existing literature on cognitive (including language) and academic performance among offspring of parents with SMIs. We further examined whether key study characteristics explain variability in effect sizes using subgroup and meta-regression analyses.

## Methods

This systematic review and meta-analysis followed the Preferred Reporting Items for Systematic Reviews and Meta-analyses (PRISMA) reporting guidelines (Zhou et al., 2024b), with the protocol registered in PROSPERO (CRD42023451106). The scope was specifically refined to focus on the offspring’s cognitive and academic performance. Deviations from the protocol are detailed in Supplementary Appendix 1.

### Eligibility criteria

We included cohort, case–control, and cross-sectional studies examining the association between parental SMIs and offspring cognitive and academic performance. Cognitive and academic performance outcomes were eligible regardless of whether they were specified as primary or secondary study outcomes. To ensure comprehensiveness and capture effects across the life course, we imposed no limits on publication year or offspring age. Studies were excluded if they were reviews, case studies, commentaries, conference abstracts, or non-English publications. However, reference lists of relevant reviews and commentaries were screened to retrieve eligible primary studies.

Parental SMI was defined as a diagnosis of schizophrenia spectrum disorders (including schizophrenia, schizoaffective disorder, and other psychotic disorders), bipolar disorder, or MDD, based on clinical records, diagnostic interviews, or validated screening tools. Studies that used the term “depression” were eligible where the underlying diagnosis or severity corresponded to MDD or severe depressive disorder. Both population-based and hospital-based studies were eligible, provided they included a clearly defined exposure and outcome with a comparison group.

### Search strategy

A comprehensive search was conducted in MEDLINE, EMBASE, PsycINFO, CINAHL, and Scopus from their inception to December 2024 and was updated in December 2025 (see Supplementary Table 1 for the full search strategy).

### Selection process

All records identified were uploaded to the Covidence platform (Veritas Health, 2025), where duplicates were removed. The first author (A.A.A.) screened titles and abstracts, with 20% undergoing double independent assessment by another author (B.D.) to assess consistency and minimize bias. This proportion is consistent with guidance from the Cochrane Handbook for Systematic Reviews, which supports partial double-screening (e.g. 10–20%) when agreement is high and resources are constrained (Higgins et al., 2024). The agreement rate for the double-screened subset was 94.8%, indicating strong reliability. To reduce the risk of excluding relevant records during the title/abstract stage, all records were evaluated against all inclusion criteria and full-text screening was conducted for any studies with unclear eligibility. Full texts were independently assessed by both reviewers using predefined criteria, with 91.8% concordance at this stage. Discrepancies were resolved through discussion. Where multiple publications were based on the same sample, the most comprehensive or most recent report was selected, unless different publications assessed offspring at distinct ages or reported non-overlapping cognitive or academic measures.

### Data extraction

The first author (A.A.A.) extracted relevant data using an Access database. Another author (A.J.) performed a double extraction of 20% of randomly selected studies and conducted an overall review to ensure accuracy and consistency. The extracted data included author, year of publication, study design, country, sample size, exposure type, exposure measurement, and exposure timing. Outcome details extracted included cognitive domain, cognitive test, and outcome assessment age (or mean age when age-specific data were unavailable). Additionally, major findings were recorded, including effect size (e.g. Cohen’s d, odds ratio), means with standard deviations (SDs), the number of exposed and unexposed participants, and covariates adjusted for. The corresponding authors were contacted for additional information.

### Risk of bias assessment

The risk of bias was assessed using the Newcastle-Ottawa Scale (NOS) (Wells et al., 2000), with modifications for cross-sectional studies (i.e. Follow-Up Period and Adequacy of Follow-Up were replaced with Response Rate and Non-Respondent Bias Assessment). Two author pairs independently rated the studies. Discrepancies were resolved through discussion.

### Statistical analysis

When mean and SD data were available, standardized mean differences (SMDs) were calculated by subtracting the mean cognitive domain score of the offspring of parents with no mental disorder from that of the offspring of parents with any of the parental SMIs divided by the pooled SD. For measures where higher scores indicate poorer performance (e.g. *Trail Making Test, Wisconsin Card Sorting Test Perseverative Errors*), the SMD was multiplied by −1 to ensure that negative values consistently reflect worse performance in the exposed group. This approach ensured consistency in interpreting cognitive measures across studies. For studies lacking mean and SD data, SMD values were estimated from other reported statistics, such as odds ratios, β coefficients, or t-test scores, using established methods (Wilson, 2023).

Offspring’s measures were categorized into nine domains: academic performance, attention, executive function, IQ, language, memory, processing speed, social cognition, and general cognition. General cognition encompasses broad cognitive performance that cannot be assigned to any of the specific subdomains. Measures included within this domain primarily reflected global or composite indices (e.g. *Bayley Scales of Infant Development*), domain-integrated subtests (e.g. *Wechsler Intelligence Scale for Children Block Design*), and a small proportion of developmental or diagnostic outcomes (e.g. *intellectual disability*). While efforts were made to ensure mutual exclusivity, some overlap may remain.

To synthesize effect sizes and explore potential sources of heterogeneity across studies, we conducted the following analyses.

First, we conducted robust meta-analyses using the *robumeta* package (Fisher, Tipton, & Zhipeng, 2017), which employs robust variance estimation to account for multiple outcomes/estimates from the same study (Hedges, Tipton, & Johnson, 2010), for each parental SMI and cognitive domain. Forest plots were generated for each parental SMI and the overall cognitive performance. Additionally, we estimated overall pooled SMDs for each parental SMI and each of the nine cognitive domains. Negative SMDs indicate worse performance in the offspring of parents with SMIs compared to controls. Second, we stratified each overall pooled estimate by affected parent (maternal vs. any parent), offspring age group (early childhood [0–5 years], childhood [6–12 years], adolescence [13–19 years], and adulthood [20+ years]), study region (North America, Europe, and Other regions), study quality (low [NOS ≤ 4], moderate [NOS 5–7], and high [NOS ≥ 8]), study design (cohort vs. cross-sectional) and covariate adjustment (unadjusted, adjusted for age/sex only, and adjusted for age/sex + other factors). The absence of studies reporting paternal SMI separately limited our comparisons to maternal versus any parent affected. Third, to explore potential sources of heterogeneity, we conducted meta-regression analyses (for each parental SMI) with robust variance estimation. Models incorporated small-sample corrections and assumed a within-study correlation (*ρ*) of 0.8 (Tipton, 2015). Each parental SMI multivariable model included parental exposure type, offspring age group, study quality, publication year (centered), and cognitive domain. Cognitive domains were treated as categorical variables, with IQ serving as the reference category, allowing estimation of domain-specific deviations from general intellectual functioning. Regression coefficients represent differences in SMD relative to reference categories within each SMI. Fourth, we conducted sensitivity analyses by excluding low-quality and the largest studies (Jundong et al., 2012; A. Lin et al., 2017b; Lin et al., 2024; Ranning et al., 2018). Fifth, we repeated the overall pooled analyses for each parental SMI after excluding studies (estimates) classified under the general cognition domain, restricting the analysis to the remaining eight specific cognitive domains. Sixth, we used funnel plots and the Egger test to assess the risk of publication bias by examining funnel plot asymmetry (Egger, Smith, Schneider, & Minder, 1997; Sterne et al., 2011). As publication bias was assessed at the study level, we used the average when multiple estimates were reported within a study. We also conducted the Precision Effect Test (PET), which regresses effect sizes on their standard errors to detect potential small-study effects (Stanley & Doucouliagos, 2014).

Between-study heterogeneity was summarized using *τ* ^2^. For all meta-analyses, we reported pooled SMDs with 95% confidence intervals (CIs), corresponding *p*-values, and *τ* ^2^. For meta-regression analyses, regression coefficients (*β*), 95% CIs, and *p*-values were reported, with *τ* ^2^ used to quantify residual heterogeneity. All statistical analyses were performed using R (R Core Team, 2024).

## Results

### Study selection

**Figure 1.**
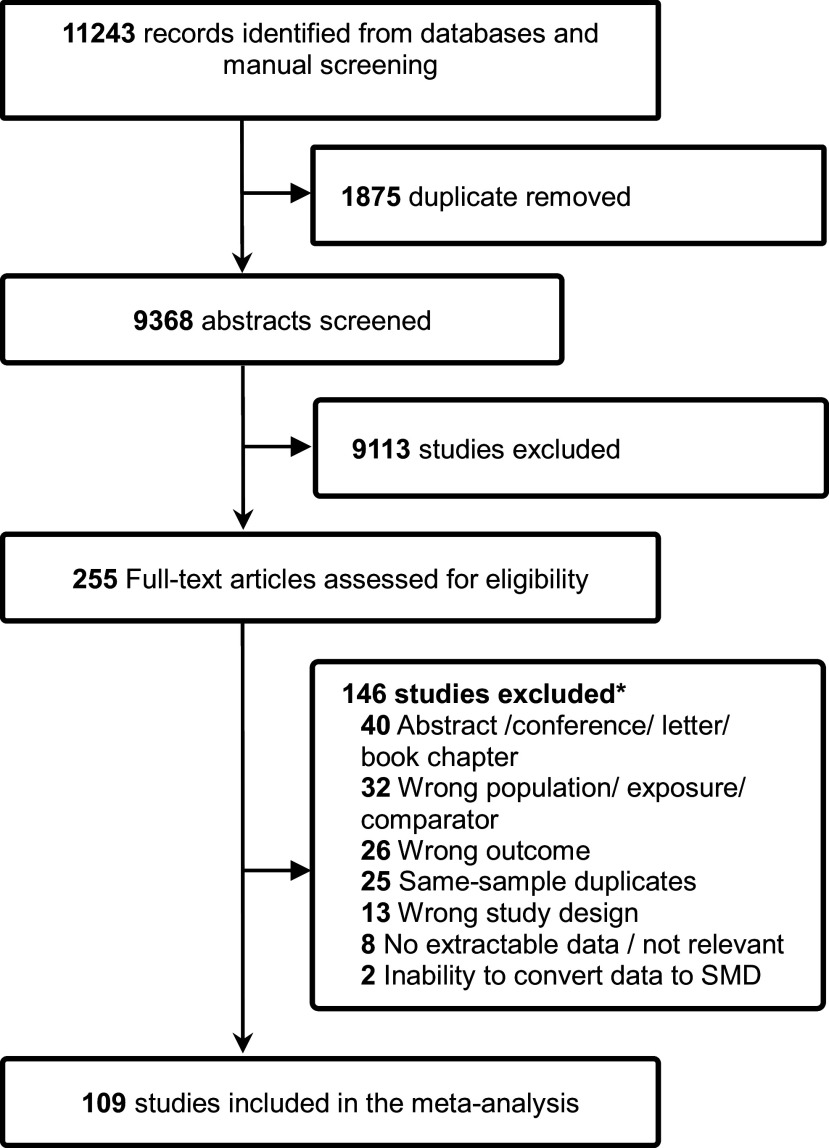
Prisma flowchart for study selection. SMD: standardised mean difference. * eTable 2 provides a list of full-text articles excluded at the eligibility stage, together with the reasons for exclusion.

A total of 11,243 records were identified through electronic databases and manual searches. After removing 1,875 duplicates and excluding 9,113 records based on title and abstract screening, 255 full-text articles were assessed for eligibility. Of these, 109 studies met the inclusion criteria. The remaining 146 articles were excluded for various reasons (e.g. wrong population or exposure, abstract only, or wrong outcomes), as shown in Figure 1. Details of excluded full-text articles and reasons for exclusion are provided in the supplementary material (Supplementary Table 2).

### Study characteristics

Most studies were cohort studies (59/109), with a strong representation from North America (51/109) and Europe (32/109). Sample sizes varied widely, ranging from 14 to 680,442 participants, contributing to a combined total of 1,586,339 participants, of which 29,671 were exposed to one or more parental SMIs, and 1160 estimates were included. MDD was the most studied parental SMI, and cognitive performance typically focused on IQ, memory, executive function, language, and general cognition.

Most studies were of moderate quality (65/109). As shown in Supplementary Table 3, parental SMIs were identified mainly through standard diagnostic systems such as the Diagnostic and Statistical Manual of Mental Disorders (DSM-III–V) and International Classification of Diseases (ICD-8–10), or structured interviews (e.g. Schedule for Affective Disorders and Schizophrenia–Lifetime Version [SADS-L], Structured Clinical Interview for DSM Disorders [SCID]). Some studies used clinician or hospital records, and a few applied validated rating tools (e.g. Hamilton Depression Rating Scale [HDRS], Patient Health Questionnaire [PHQ]). Overall, most studies relied on established, reliable diagnostic methods to ascertain parental SMIs.

Similarly, offspring cognitive and academic performance were measured using standardized, validated instruments, including the Wechsler Scales for IQ, Wisconsin Card Sorting Test (WCST) and Trail Making Test B for executive function, Continuous Performance Test (CPT) and Test of Variables of Attention (TOVA) for attention, Wechsler Memory Scale (WMS) and California Verbal Learning Test (CVLT) for memory, and school-based measures for academic performance (further details available in Supplementary Tables 4a–c and Supplementary Table 5). Table 1 summarizes key study characteristics and mean SMD in cognitive performance across parental SMIs.

**Table 1.** Descriptive summary of studies included in the systematic review and meta-analyses

Characteristics	Schizophrenia *N = 1,410,156*		Bipolar disorder *N = 778,934*		Major depressive disorder *N = 267,510*	
	No. of studies (Estimates)	Mean SMD (SE)	No. of studies (Estimates)	Mean SMD (SE)	No. of studies (Estimates)	Mean SMD (SE)
Overall	45 (450)	−0.58 (0.05)	44 (451)	−0.32 (0.03)	51 (259)	−0.18 (0.03)
Age group, years						
Early childhood (0–5)	6 (18)	−0.76 (0.26)	2 (7)	−0.08 (0.03)	23 (80)	−0.23 (0.06)
Childhood (6–12)	26 (273)	−0.73 (0.07)	25 (241)	−0.21 (0.04)	20 (118)	−0.14 (0.04)
Adolescence (13–19)	12 (70)	−0.36 (0.05)	18 (158)	−0.39 (0.06)	10 (56)	−0.21 (0.06)
Adulthood (20+)	4 (89)	−0.25 (0.03)	2 (45)	−0.76 (0.15)	1 (5)	−0.15 (0.06)
Cognitive domain						
Academic performance	5 (12)	−0.30 (0.12)	4 (13)	−0.26 (0.09)	4 (15)	−0.42 (0.10)
Attention	14 (54)	−0.48 (0.13)	13 (60)	0.04 (0.07)	7 (35)	−0.04 (0.09)
Executive function	17 (142)	−0.39 (0.03)	20 (163)	−0.40 (0.06)	8 (36)	−0.08 (0.03)
Memory	14 (86)	−0.88 (0.16)	15 (86)	−0.48 (0.08)	7 (24)	−0.20 (0.13)
Language	14 (32)	−0.65 (0.17)	7 (15)	−0.16 (0.09)	19 (42)	−0.20 (0.05)
Processing speed	10 (25)	−0.85 (0.34)	11 (38)	−0.35 (0.18)	3 (17)	−0.12 (0.08)
IQ	18 (30)	−0.53 (0.07)	20 (28)	−0.39 (0.07)	15 (21)	−0.28 (0.06)
Social cognition	6 (42)	−0.26 (0.04)	5 (22)	−0.04 (0.09)	4 (15)	−0.25 (0.09)
General cognition	10 (27)	−1.13 (0.25)	10 (26)	−0.44 (0.14)	24 (54)	−0.22 (0.08)
Affected by SMI						
Mother	9 (93)	−0.30 (0.06)	6 (35)	−0.28 (0.05)	35 (161)	−0.17 (0.04)
Any parent	36 (357)	−0.65 (0.06)	38 (416)	−0.33 (0.04)	17 (98)	−0.19 (0.03)
Publication period						
2020–2025	15 (194)	−0.90 (0.10)	14 (189)	−0.28 (0.06)	8 (34)	−0.26 (0.05)
2010–2019	14 (98)	−0.39 (0.03)	22 (188)	−0.35 (0.05)	21 (64)	−0.22 (0.08)
2000–2009	8 (134)	−0.28 (0.03)	5 (66)	−0.40 (0.05)	13 (141)	−0.13 (0.03)
Before 2000	8 (24)	−0.38 (0.10)	3 (8)	−0.15 (0.05)	9 (20)	−0.28 (0.10)
Study region						
Europe	20 (265)	−0.67 (0.07)	16 (174)	−0.27 (0.07)	10 (26)	−0.22 (0.05)
North America	13 (83)	−0.29 (0.04)	20 (128)	−0.38 (0.04)	29 (183)	−0.15 (0.03)
Other Regions	12 (102)	−0.58 (0.06)	8 (149)	−0.33 (0.05)	12 (50)	−0.27 (0.06)
Study quality						
Low	6 (35)	−0.88 (0.12)	7 (61)	−0.62 (0.12)	4 (18)	−0.41 (0.17)
Moderate	26 (338)	−0.61 (0.06)	26 (310)	−0.31 (0.04)	26 (154)	−0.15 (0.04)
High	13 (77)	−0.30 (0.03)	11 (80)	−0.16 (0.03)	21 (87)	−0.19 (0.03)
Study design						
Cross-sectional	19 (121)	−0.51 (0.04)	22 (215)	−0.32 (0.04)	16 (54)	−0.25 (0.06)
Cohort	26 (329)	−0.61 (0.06)	22 (236)	−0.32 (0.05)	35 (205)	−0.16 (0.03)
Covariate adjustment						
Unadjusted	22 (167)	−0.34 (0.02)	26 (213)	−0.21 (0.02)	35 (174)	−0.19 (0.03)
Age/sex only	8 (163)	−0.31 (0.03)	9 (95)	−0.35 (0.04)	5 (15)	−0.30 (0.11)
Age/sex + other factors	15 (120)	−1.27 (0.15)	9 (143)	−0.48 (0.10)	11 (70)	−0.14 (0.05)

IQ, intelligence quotient; SE, standard error; SMD, standardized mean difference.

### Cognitive performance in offspring of parents with SMIs

**Figure 2.**
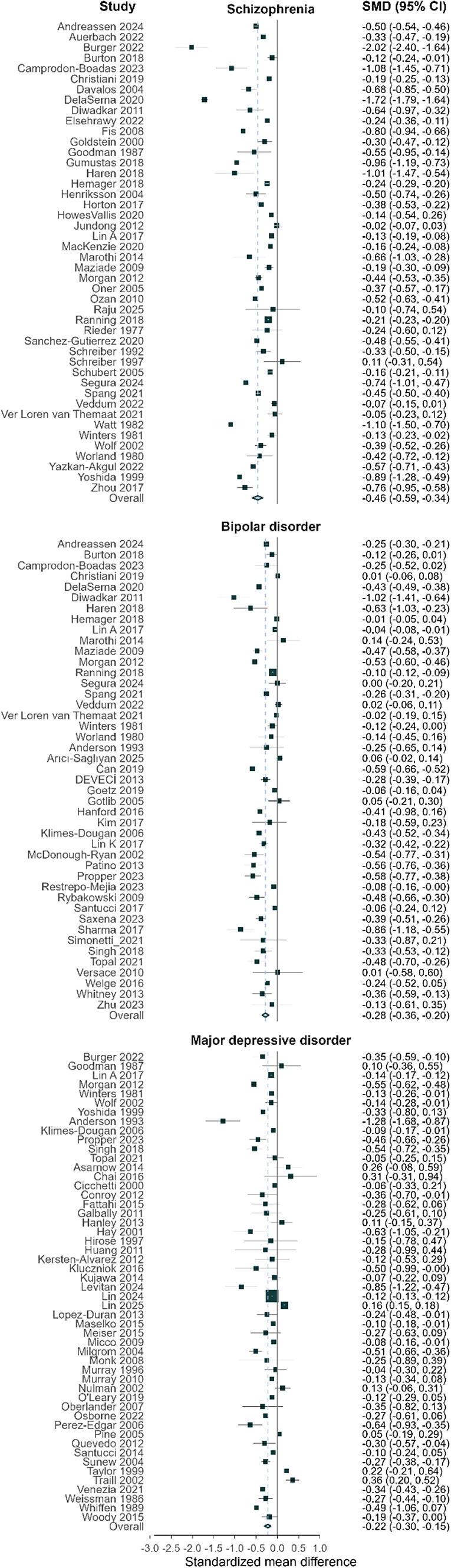
Forest plot of the association between parental severe mental illness and offspring overall cognitive outcomes. Effect sizes were aggregated per study using inverse-variance weighting. A diamond at the bottom of each plot indicates the robust pooled estimate based on a robust variance estimation model. Positive values reflect better cognitive performance among exposed offspring, while negative values reflect worse performance relative to comparison groups.

Table 2 shows pooled effect sizes by cognitive domain and parental SMI type, while Figure 2 displays a forest plot of the associations between parental SMI and overall offspring cognitive performance. The overall cognitive performance of offspring of parents with schizophrenia was significantly poorer than that of offspring of parents without mental illness (SMD = −0.46, 95% CI: −0.59, −0.34), with moderate heterogeneity between studies (τ^2^ = 0.083). When compared to controls, offspring of parents with schizophrenia exhibited poorer cognitive performance across most domains, including general cognition (SMD = −1.07, 95% CI: −1.92, −0.22), language (SMD = −0.70, 95% CI: −1.20, −0.20), IQ (SMD = −0.53, 95% CI: −0.72, −0.34), memory (SMD = −0.58, 95% CI: −1.08, −0.09), attention (SMD = −0.33, 95% CI: −0.59, −0.07), and executive function (SMD = −0.34, 95% CI: −0.52, −0.17). Associations with other cognitive domains and academic performance were not statistically significant.

**Table 2.** Effect sizes of cognitive outcomes in children of parents with severe mental illness

Cognitive domain	No. of exposed	No. of controls	SMD (95% CI)	*p*-value	*τ* ^2^
*Schizophrenia*					
Academic performance	5849	1394731	−0.17 (−0.47, 0.13)	0.175	0.036
Attention	943	1217	−0.33 (−0.59, −0.07)	0.015	0.226
Executive function	1248	1622	−0.34 (−0.52, −0.17)	0.001	0.124
IQ	1134	1320	−0.53 (−0.72, −0.34)	<0.001	0.086
Language	974	1149	−0.70 (−1.20, −0.20)	0.01	0.364
Memory	809	1102	−0.58 (−1.08, −0.09)	0.024	0.440
Processing speed	530	793	−1.00 (−2.07, 0.07)	0.064	0.874
Social cognition	493	513	−0.30 (−0.61, 0.01)	0.055	0.057
General cognition	1037	3910	−1.07 (−1.92, −0.22)	0.019	0.485
*Overall*	*9114*	*1401042*	*−0.46 (−0.59, −0.34)*	*<0.001*	*0.083*
*Bipolar disorder*					
Academic performance	4618	764962	−0.16 (−0.55, 0.23)	0.218	0.026
Attention	738	1037	−0.11 (−0.31, 0.09)	0.242	0.175
Executive function	1063	1434	−0.34 (−0.51, −0.16)	0.001	0.347
IQ	876	1100	−0.32 (−0.48, −0.17)	<0.001	0.054
Language	543	765	−0.18 (−0.34, −0.02)	0.034	0.116
Memory	738	1009	−0.40 (−0.60, −0.19)	0.001	0.374
Processing speed	573	711	−0.57 (−1.42, 0.28)	0.165	1.178
Social cognition	327	464	−0.02 (−0.26, 0.22)	0.818	0.051
General cognition	1966	4018	−0.45 (−0.79, −0.12)	0.013	0.202
*Overall*	*7995*	*770939*	*−0.28 (−0.36, −0.20)*	*0 < 0.001*	*0.088*
*Major depressive disorder*					
Academic performance	1862	88489	−0.46 (−1.25, 0.33)	0.161	0.203
Attention	447	569	−0.18 (−0.65, 0.28)	0.374	0.165
Executive function	501	597	−0.20 (−0.39, −0.01)	0.045	0.024
IQ	1113	892	−0.23 (−0.37, −0.08)	0.006	0.016
Language	16568	153497	−0.12 (−0.23, −0.02)	0.022	0.032
Memory	525	373	−0.20 (−0.41, 0.00)	0.052	0.194
Processing speed	163	114	−0.25 (−0.90, 0.41)	0.247	0.056
Social cognition	179	150	−0.15 (−0.63, 0.33)	0.383	0.106
General cognition	17953	156192	−0.22 (−0.34, −0.11)	<0.001	0.082
*Overall*	*20980*	*246530*	*−0.22 (−0.30, −0.15)*	<0.001	*0.063*

CI, confidence interval; IQ, intelligence quotient; SMD, standardized mean difference.

The overall cognitive performance of offspring of parents with bipolar disorder was significantly poorer than that of offspring of parents without mental illness (SMD = −0.28, 95% CI: −0.36, −0.20), with moderate heterogeneity between studies (*τ* ^2^ = 0.088). The offspring of parents with bipolar disorder exhibited lower performance in general cognition (SMD = −0.45, 95% CI: −0.79, −0.12), memory (SMD = −0.40, 95% CI: −0.60, −0.19), executive function (SMD = −0.34, 95% CI: −0.51, −0.16), language (SMD = −0.18, 95% CI: −0.34, −0.02), and IQ (SMD = −0.32, 95% CI: −0.48, −0.17), compared to controls. No statistically significant associations were found for academic performance, attention, social cognition, or processing speed.

The overall cognitive performance of offspring of parents with MDD was significantly worse than that of offspring of parents without mental illness (SMD = −0.22, 95% CI: −0.30, −0.15), with moderate heterogeneity between studies (*τ* ^2^ = 0.063). Compared to controls, offspring of parents with MDD showed worse performance in three cognitive domains: IQ (SMD = −0.23, 95% CI: −0.37, −0.08), general cognition (SMD = −0.22, 95% CI: −0.34, −0.11) and language skill (SMD = −0.12, 95% CI: −0.23, −0.02). No statistically significant associations were observed for academic performance, attention, executive function, memory, social cognition, or processing speed.

### Subgroup and meta-regression analyses

Table 3 presents the overall pooled effect sizes for each parental SMI, stratified by key study characteristics. Among offspring of parents with schizophrenia, estimates were broadly consistent across exposure type, age group, study design, covariate adjustment, and region, with the exception of study quality, where larger effects were observed in low- and moderate-quality studies compared with high-quality studies. Among offspring of parents with bipolar disorder, slightly stronger associations were noted during adolescence and in studies with lower or moderate quality, whereas associations for maternal exposure and early childhood or adulthood were not statistically significant. For MDD, associations were largely consistent across subgroups, with slightly greater associations in low-quality studies.

**Table 3.** Effect sizes of overall cognitive outcomes in children of parents with severe mental illness, stratified by key study characteristics

Subgroup	No. of exposed	No. of controls	SMD (95% CI)	*P*-value	*τ* ^2^
*Schizophrenia*					
Exposure					
Maternal	1206	91796	−0.53 (−0.90, −0.16)	0.012	0.097
Parental	7908	1309246	−0.45 (−0.59, −0.31)	0.000	0.090
Age group, years					
Early childhood (0–5)	240	285	−0.66 (−1.55, 0.23)	0.116	0.519
Childhood (6–12)	2980	93895	−0.47 (−0.65, −0.29)	0.000	0.131
Adolescence (13–19)	5822	1306779	−0.39 (−0.65, −0.14)	0.007	0.054
Adulthood (20+)	176	303	−0.43 (−0.82, −0.04)	0.038	0.140
Study quality					
Low	305	309	−0.87 (−1.57, −0.17)	0.024	0.441
Moderate	1523	1925	−0.53 (−0.73, −0.33)	0.000	0.257
High	7286	1398808	−0.27 (−0.40, −0.13)	0.001	0.039
Study design					
Cross-sectional	2301	630586	−0.49 (−0.67, −0.32)	0.000	0.174
Cohort	6813	770456	−0.46 (−0.64, −0.27)	0.000	0.100
Covariate adjustment					
No adjustment	2076	2329	−0.38 (−0.50, −0.26)	0.000	0.070
Age/sex only	222	315	−0.43 (−0.66, −0.20)	0.003	0.130
Age/sex + other	6816	1398398	−0.60 (−0.95, −0.25)	0.003	0.090
Study region					
Europe	7063	1308272	−0.45 (−0.68, −0.22)	0.001	0.088
North America	828	937	−0.40 (−0.58, −0.23)	0.000	0.095
Other regions	1223	91833	−0.57 (−0.86, −0.29)	0.001	0.110
*Bipolar disorder*					
Exposure					
Maternal	2195	91705	−0.22 (−0.51, 0.08)	0.115	0.108
Parental	5800	679234	−0.30 (−0.39, −0.21)	0.000	0.123
Age group, years					
Early childhood (0–5)	149	208	−0.20 (−1.32, 0.92)	0.266	0.000
Childhood (6–12)	3539	93640	−0.23 (−0.32, −0.13)	0.000	0.136
Adolescence (13–19)	4398	677235	−0.37 (−0.53, −0.21)	0.000	0.291
Adulthood (20+)	104	100	−0.64 (−2.47, 1.19)	0.140	1.091
Study quality					
Low	293	334	−0.32 (−0.60, −0.05)	0.029	0.269
Moderate	1330	1752	−0.33 (−0.45, −0.21)	0.000	0.232
High	6372	768853	−0.17 (−0.32, −0.02)	0.029	0.059
Study design					
Cross-sectional	770	856	−0.32 (−0.45, −0.19)	0.000	0.165
Cohort	7225	770083	−0.25 (−0.36, −0.14)	0.000	0.083
Covariate adjustment					
No adjustment	1531	2289	−0.21 (−0.31, −0.12)	0.000	0.046
Age/sex only	290	317	−0.31 (−0.46, −0.17)	0.001	0.131
Age/sex + other	6174	768333	−0.41 (−0.68, −0.14)	0.008	0.114
Study region					
Europe	5000	678246	−0.22 (−0.36, −0.07)	0.007	0.134
North America	693	956	−0.34 (−0.46, −0.23)	0.000	0.094
Other Regions	2302	91737	−0.30 (−0.54, −0.06)	0.020	0.121
*Major depressive disorder*					
Exposure					
Maternal	4887	93840	−0.24 (−0.34, −0.14)	0.000	0.088
Parental	16093	152616	−0.17 (−0.30, −0.04)	0.013	0.047
Age group, years					
Early childhood (0–5)	15996	153347	−0.18 (−0.29, −0.08)	0.002	0.043
Childhood (6–12)	11956	168678	−0.21 (−0.38, −0.05)	0.013	0.119
Adolescence (13–19)	660	393	−0.24 (−0.37, −0.11)	0.002	0.133
Adulthood (20+)	NA				
Study quality					
Low	151	215	−0.39 (−0.73, −0.04)	0.038	0.622
Moderate	1262	1350	−0.25 (−0.38, −0.12)	0.000	0.130
High	19567	244965	−0.18 (−0.28, −0.07)	0.003	0.056
Study design					
Cross-sectional	1039	1164	−0.23 (−0.31, −0.14)	0.000	0.096
Cohort	19941	245366	−0.22 (−0.32, −0.12)	0.000	0.062
Covariate adjustment					
No adjustment	2204	2562	−0.24 (−0.32, −0.15)	0.000	0.074
Age/sex only	283	173	−0.19 (−0.61, 0.23)	0.261	0.099
Age/sex + other	18493	243795	−0.19 (−0.40, 0.02)	0.074	0.061
Study region					
Europe	369	510	−0.27 (−0.42, −0.13)	0.003	0.011
North America	1607	2043	−0.21 (−0.33, −0.09)	0.001	0.116
Other Regions	19004	243977	−0.21 (−0.38, −0.04)	0.023	0.060

CI, confidence interval; NA, not sufficient data available; SMD, standardized mean difference.

*Note:* The stratified analysis includes any cognitive domains (detailed in Table 2) assessed across the studies.

Results from the multivariable meta-regression analyses are presented in Supplementary Table 6. Parental exposure type was not associated with offspring cognitive performance in any disorder. Offspring age-related effects were limited. In bipolar disorder, adult offspring showed significantly poorer cognitive performance compared with early childhood (*β* = −0.73, 95% CI: −1.36, −0.10), while no significant age differences were observed in schizophrenia or MDD. Study quality was a significant moderator in schizophrenia, with larger deficits reported in low (*β* = −0.63, 95% CI: −1.05, −0.21) and moderate (*β* = −0.24, 95% CI: −0.45, −0.03) quality studies compared with high-quality studies. A similar but weaker pattern was observed for MDD. Publication year was inversely associated with effect size in schizophrenia only (*β* = −0.01, 95% CI: −0.02, −0.00), indicating smaller deficits in more recent studies. After adjustment for study-level characteristics, domain-specific effects were largely confined to schizophrenia, where several cognitive domains showed significantly better performance relative to IQ. In contrast, no cognitive domain differed significantly from IQ in the bipolar disorder or MDD model.

### Sensitivity and publication bias analyses

Sensitivity analyses excluding large, linked data studies (primarily academic performance studies) produced results consistent with the main analysis (Supplementary Table 7). Similarly, the exclusion of low-quality studies did not meaningfully alter the findings. Nonetheless, associations for the language domain across all parental SMIs, and for general cognition in schizophrenia, were modestly attenuated and spanned the null value (Supplementary Table 8). Repeating the overall analyses after excluding the general cognition domain did not meaningfully alter the pattern or direction of associations across the remaining eight cognitive and academic measures (Supplementary Figure 1).

Visual inspection of the funnel plots suggested possible publication bias (Supplementary Figure 2). Egger’s test indicated a significant funnel plot asymmetry for studies of offspring of parents with schizophrenia (*p* = 0.005), but not for bipolar disorder (*p* = 0.134) or MDD (*p* = 0.217) (Supplementary Table 9). Consistent with these results, meta-regression analyses examining the association between effect sizes and their standard errors indicated significant small-study effects for schizophrenia (*β* = −2.18, *p* = 0.032), borderline evidence for bipolar disorder (*β* = −1.16, *p* = 0.050), and no evidence of small-study effects for MDD (*p* = 0.136) (Supplementary Table 10).

## Discussion

In this comprehensive meta-analysis, we found that the offspring of parents with SMIs consistently exhibited reduced performance across various cognitive domains compared to the offspring of parents without mental illness. Using conventional Cohen’s *d* thresholds (small = 0.2, moderate = 0.5, large = 0.8), we observed moderate-to-large effect sizes for the offspring of parents with schizophrenia, small-to-moderate effect sizes for those with parental bipolar disorder, and small effect sizes for those with parental MDD. This differential impact could be due to varying severity, chronicity, and neurodevelopmental effects associated with each parental SMI.

The strength of evidence varied across cognitive domains. For example, among offspring of parents with schizophrenia, several domains, including IQ, executive function, attention, memory, and language, showed consistent negative associations and were based on 10 or more studies, highlighting the robustness of the evidence. Similarly, the domains showing significant associations among offspring of parents with bipolar disorder and MDD were generally supported by an adequate number of studies. However, the relatively large τ^2^ values observed in several domains suggest substantial heterogeneity across studies, which may reflect differences in cognitive assessments, developmental stages at testing, and other study characteristics.

Parental SMIs were not significantly associated with all cognitive domains. For instance, schizophrenia and bipolar disorder showed no associations with academic performance or processing speed, while MDD was not significantly linked to most domains. These null findings may reflect limited statistical power or true domain-specific variability. Nonetheless, most estimates were negative and suggestive of possible associations, warranting further investigation.

Parental SMIs can influence offspring’s cognitive development through a complex interplay of genetic and environmental factors. Genetically, the inheritance of vulnerability genes may increase the risk of cognitive impairments in children (Gottesman & Gould, 2003; Toulopoulou et al., 2010). From an environmental perspective, children of parents with SMIs are more likely to experience disrupted caregiving, chronic stress, and lower socioeconomic status, all of which are well-established risk factors for impaired cognitive development (Kamis, 2021; Krantz et al., 2023; Lupien, McEwen, Gunnar, & Heim, 2009). Schizophrenia, considered the most debilitating and chronic of these conditions (Global Burden of Disease Collaborators, 2018; Zhu et al., 2019) is associated with the most pronounced cognitive impact on offspring. Additionally, children of parents with SMIs are more likely to experience early life adversity, which can have long-term effects on brain development, particularly in regions involved in executive function, memory, and attention (Aktar et al., 2019; Hussain, 2020; McLaughlin, Sheridan, & Lambert, 2014). These cognitive challenges often translate into academic difficulties and behavioral regulation issues, with potentially lasting consequences throughout childhood and adolescence (Blok et al., 2023; Peng & Kievit, 2020).

Our meta-regression analyses indicated that offspring age moderated the differences in overall cognitive performance between offspring of parents with bipolar disorder and controls. Cognitive deficits were more pronounced in adolescence and adulthood in the bipolar disorder model, suggesting that cognitive differences may become more apparent as children grow older and cognitive demands increase. This pattern may also reflect gene–environment interactions, prolonged exposure to parental symptoms, and increasing developmental and environmental demands. The episodic nature of bipolar disorder can create a chronically unstable home environment, impairing emotional regulation and executive functioning in offspring over time (Duffy, Vandeleur, Heffer, & Preisig, 2017). However, because the present analyses are based primarily on cross-sectional comparisons across age groups rather than longitudinal follow-up within the same individuals, these interpretations should be made cautiously, and the findings need to be replicated in prospective longitudinal studies. In the schizophrenia model, low study quality was associated with the greatest deficit in cognitive performance between offspring of parents with schizophrenia and controls. Notably, this finding is primarily based on few small-scale studies (*n* = 6) and may reflect greater sampling variability as well as potential publication bias (i.e. small-study effects) (Schwarzer, Carpenter, & Rücker, 2015). Supporting this interpretation, PET indicated significant evidence of small-study effect for schizophrenia studies and borderline evidence for bipolar disorder, but no evidence of small-study effects for MDD, suggesting that such effects may be particularly relevant for schizophrenia literature. However, sensitivity analyses excluding these studies did not materially alter conclusions, supporting the robustness of our findings. Additionally, meta-regression analyses indicated that the cognitive domain significantly moderated the overall cognitive performance difference between offspring of parents with schizophrenia and controls, whereas no such moderation was observed for bipolar disorder or MDD. Relative to the IQ domain, several domains, including academic performance, attention, executive function, language, and social cognition, showed significantly smaller deficits. This pattern may suggest that general intellectual functioning is particularly sensitive to familial risk for schizophrenia and is consistent with the hypothesis that lower IQ represents an early neurodevelopmental marker of schizophrenia risk, as reduced intellectual functioning has been consistently observed in individuals who later develop schizophrenia and in their unaffected relatives (Dickson, Laurens, Cullen, & Hodgins, 2012; Woodberry, Giuliano, & Seidman, 2008).

Collectively, the findings suggest the potential importance of early, preventive care through integrated, family-centered services that coordinate health, social, and educational supports that may help mitigate cognitive developmental risk and its long-term costs (Center on the Developing Child at Harvard University, 2016). Perinatal parent–infant mental health programs have been shown in previous research to enhance caregiving quality and reduce early stress, while developmental screening for children of parents with SMI may facilitate earlier identification of developmental concerns and intervention (Glascoe, 2000; Stein et al., 2014). Wrap-around models, such as home visiting, aim to address the needs of both parent and child simultaneously, relying on collaboration across primary care, mental health, child health, and early education (Olds et al., 2007; World Health, 2021). These approaches may also benefit from policies that recognize parental mental illness as an early-childhood risk factor, alongside robust data systems for early detection, and investment in high-quality early childhood education with embedded cognitive, behavioral, and emotional supports (Maybery & Reupert, 2009; Melhuish et al., 2015).

### Strengths and limitations

To the best of our knowledge, this is the first comprehensive meta-analysis to examine the association between parental SMIs, including schizophrenia, bipolar disorder, and MDD, and a range of offspring academic and cognitive performance domains, drawing on data from over 1.5 million participants. The inclusion of studies without restrictions on publication year strengthens the robustness of our findings by incorporating both historical and contemporary evidence. The large sample size provided substantial statistical power, enabling detailed subgroup and meta-regression analyses to explore the source of variations. Additionally, applying robust meta-analytic methods with variance estimation allowed us to account for correlation bias arising from multiple estimates within individual studies. Most included studies also employed consistent diagnostic approaches for each parental SMI, which supports the validity and comparability of exposure definitions across studies.

This review has several limitations. There was substantial heterogeneity in study design, sample size, study quality, and cognitive measures, which can contribute to variability in effect estimates and limit comparability. Most studies were small in scale, and certain domains, particularly academic performance, were underrepresented. These gaps highlight the need for more high-quality research, especially focusing on academic performance and distinct types of parental SMI. Potential publication and residual confounding biases cannot be entirely ruled out, though subgroup analyses adjusting for key covariates (e.g. offspring age, sex, and parental SES) yielded consistent findings. The exclusion of non-English studies and the predominance of research from high-income countries may limit generalizability. Finally, due to limited data on paternal SMI, we could not compare maternal and paternal effects, underscoring the need for more inclusive research to inform family-based interventions.

## Conclusion

This systematic review and meta-analysis of over 1.5 million offspring–parent pairs found consistent and robust negative associations between parental SMIs and offspring cognitive performance from childhood to adulthood. The strongest associations were observed for parental schizophrenia, whereas associations were generally weaker for parental MDD. For parental bipolar disorder, offspring cognitive deficits appeared to be most pronounced in adulthood, while for schizophrenia, the cognitive domain significantly moderated the association, with IQ showing the largest deficit. Collectively, these findings underscore the importance of early integrated, wrap-around interventions targeting families affected by SMI, with the potential to support population-level enhancements in cognitive development and broader health outcomes.

## Supporting information

10.1017/S0033291726103985.sm001 Adane et al. supplementary material Adane et al. supplementary material

## Data Availability

A.A.A. and A.J. have full access to all data in the study and takes responsibility for the integrity of the data and the accuracy of the data analyses. The data that support the findings of this study and/or codes are available on request.

## Supplementary Material

The supplementary material for this article can be found at http://doi.org/10.1017/S0033291726103985.
