# Supplementary material for: Cognitive performance in offspring of parents with severe mental illness: a meta-analysis
Source: Psychol Med. 2026 Apr 27;56:e115. doi: 10.1017/S0033291726103985 (PMC13125935; doi:10.1017/S0033291726103985)
Supplement: Adane et al. supplementary material [file S0033291726103985sup001.pdf]

Online Supplemental Files

Cognitive Performance in Offspring of Parents with Severe Mental Illness: A Meta-Analysis

Contents

eAppendix 1: Deviations from the Registered Protocol (PROSPERO: *CRD42023451106*)....................2

eTable 1. Search strategies and key terms 3

eTable 2. Full-text articles excluded and reasons for exclusion. 4

eTable 3. Diagnostic methods for parental severe mental illness in included studies....................5

eTable 4a. Specific cognitive measures and corresponding domains in studies of parental schizophrenia and offspring cognitive outcomes. 7

eTable 4b. Specific cognitive measures and corresponding domains in studies of parental bipolar disorder and offspring cognitive outcomes. 14

eTable 4c. Specific cognitive measures and corresponding domains in studies of parental major depressive disorder and offspring cognitive outcomes....................20

eTable 5. Summary of cognitive measure (subdomains) included in the general cognition domain ..24

eTable 6. Meta-regression of study-level characteristics and cognitive domains on offspring cognitive performance 26

eTable 7. Effect sizes of cognitive outcomes in children of parents with severe mental illness, excluding large studies....................27

eTable 8. Effect sizes of cognitive outcomes in children of parents with severe mental illness, excluding low-quality studies as determined by the Newcastle-Ottawa Scale....................27

eFigure 1. Forest plot of the association between parental severe mental illness and offspring overall cognitive outcomes after exclusion of the general cognition domain. 29

eFigure 2. Funnel plots of studies included in the meta-analysis of parental severe mental illness and offspring cognitive outcomes, based on the mean effect estimate per study....................30

eTable 9. Egger's Test for Funnel Plot Asymmetry Based on Mean Estimates per Study....................30

eTable 10. Publication bias test using PET regression approach. 30

References 30

eAppendix 1: Deviations from the Registered Protocol (PROSPERO: *CRD42023451106*)

The following deviations from the registered protocol were made during the course of the systematic review. These changes were necessitated by methodological refinements, the evolving scope of the review, and practical considerations encountered during implementation. As the protocol had already been published, amendments could not be registered retrospectively. However, all deviations are transparently documented below.

1. Authorship Changes
Since the time of protocol registration, there have been changes in the composition of the authorship team:

- **No longer involved:** RM, MG, and HB
- **Newly added co-authors:** AJ, AWT, and BBA

These changes reflect evolving contributions and responsibilities throughout the review process and are in accordance with authorship guidelines for systematic reviews.

2. Refinement of Child Outcomes
The original protocol proposed a broad focus on a range of child outcomes, including physical health indicators (e.g., injury, infection, chronic conditions, hospitalisation, and mortality) as well as multiple developmental domains (motor, cognitive, language, and socio-emotional development).

During the initial stages of the review, it became evident that this scope was too broad to allow for meaningful synthesis and interpretation. Accordingly, a decision was made to refine the focus to offspring's cognitive and academic outcomes. This narrowing of scope enabled more rigorous evaluation of the evidence base while maintaining alignment with the overarching research aims. The specified parental exposures (schizophrenia, bipolar disorder, and major depressive disorder) remained unchanged.

3. Inclusion of Cross-Sectional Studies
As stated in the protocol, the review was initially restricted to longitudinal study designs (i.e., cohort and case-control studies). However, after refining the outcome scope, the number of eligible longitudinal studies was anticipated to be limited. To enhance the comprehensiveness of the review, and in line with best practices for evidence synthesis, the inclusion criteria were expanded to allow for the inclusion of analytically robust cross-sectional studies that met all other eligibility criteria. Subgroup analyses were conducted to examine whether study design (cohort vs cross-sectional) influenced the findings. This adjustment improved the generalisability and interpretability of the results while remaining methodologically sound.

4. Screening and Data Management Procedures
The original protocol stated that EndNote would be used for citation management and that all records would be independently screened by two reviewers at each stage—title, abstract, and full text. During the actual review, we adopted Covidence, a dedicated platform for systematic reviews, which integrates the title and abstract screening stages into a single step. Due to resource constraints and the large volume of retrieved records, the screening strategy was modified as follows:

- A single reviewer conducted the combined title and abstract screening.
- A randomly selected 20% subset of the records was independently screened by a second reviewer to assess consistency.
- Full-text screening was conducted by two independent reviewers, with discrepancies resolved through discussion or adjudication.

This modified screening approach aligns with accepted standards in systematic review methodology. Sole-reviewer screening at the title and abstract stage—even without random double screening—is widely accepted, including in the Cochrane Handbook and by high-impact journals.

eTable 1. Search strategies and key terms

S no	Search Terms
MEDLINE	
1	parent* OR maternal OR paternal OR mother* OR father*
2	"severe mental illness" OR “serious mental illness” OR "severe mental health" OR “severe psychiatric disorder” OR schizophrenia OR psychosis OR psychotic OR mania OR “bipolar disorder” OR “bipolar disease” OR “major depressive disorder" OR "depressive disorder" OR "major depression"
3	child OR children OR offspring OR adolescent* OR youth OR infant OR pediatric OR teenager
4	cognit* OR IQ OR intelligen* OR neurodevelopment OR “mental development” OR neuropsych* OR “school performance” OR academic OR education OR learning OR “child development” OR “language development” OR “developmental vulnerability” OR "developmental delay" OR “developmental risk”
5	1 AND 2 AND 3 AND 4
6	Limit #5 to (English language and humans)
EMBASE	
1	parent* OR maternal OR paternal OR mother* OR father*
2	"severe mental illness" OR “serious mental illness” OR "severe mental health" OR “severe psychiatric disorder” OR schizophrenia OR psychosis OR psychotic OR mania OR “bipolar disorder” OR “bipolar disease” OR “major depressive disorder" OR "depressive disorder" OR "major depression"
3	child OR children OR offspring OR adolescent* OR youth OR infant OR pediatric OR teenager
4	cognit* OR IQ OR intelligen* OR neurodevelopment OR “mental development” OR neuropsych* OR “school performance” OR academic OR education OR learning OR “child development” OR “language development” OR “developmental vulnerability” OR "developmental delay" OR “developmental risk”
5	1 AND 2 AND 3 AND 4
6	Limit #5 to (English language and humans)
PSYCINFO	
1	parent* OR maternal OR paternal OR mother* OR father*
2	"severe mental illness" OR “serious mental illness” OR "severe mental health" OR “severe psychiatric disorder” OR schizophrenia OR psychosis OR psychotic OR mania OR “bipolar disorder” OR “bipolar disease” OR “major depressive disorder" OR "depressive disorder" OR "major depression"
3	child OR children OR offspring OR adolescent* OR youth OR infant OR pediatric OR teenager
4	cognit* OR IQ OR intelligen* OR neurodevelopment OR “mental development” OR neuropsych* OR “school performance” OR academic OR education OR learning OR “child development” OR “language development” OR “developmental vulnerability” OR "developmental delay" OR “developmental risk”
5	1 AND 2 AND 3 AND 4
6	Limit #5 to (English language and humans)
CINAHL	
1	parent* OR maternal OR paternal OR mother* OR father*
2	"severe mental illness" OR “serious mental illness” OR "severe mental health" OR “severe psychiatric disorder” OR schizophrenia OR psychosis OR psychotic OR mania OR “bipolar disorder” OR “bipolar disease” OR “major depressive disorder" OR "depressive disorder" OR "major depression"
3	child OR children OR offspring OR adolescent* OR youth OR infant OR pediatric OR teenager
4	cognit* OR IQ OR intelligen* OR neurodevelopment OR “mental development” OR neuropsych* OR “school performance” OR academic OR education OR learning OR “child development” OR “language development” OR “developmental vulnerability” OR "developmental delay" OR “developmental risk”
5	1 AND 2 AND 3 AND 4
6	Limit #5 to (English language)

eTable 2. Full-text articles excluded and reasons for exclusion.

Study	Reason for exclusion	Study	Reason for exclusion
Andreassen 2023[1]	Duplicate of Hemager 2018 & not extractable data	Hemager 2022[2]	Duplicate of Hemager 2018
Auerbach 1993[3]	Wrong outcome	Henin 2005[4]	Wrong outcome
Ayano 2023[5]	No specific parental SMI data	Ingstrup 2019[6]	Research letter
Bakshi 2011[7]	Descriptive- Brain imaging study	Jerlang-Christiani 2015[8]	Abstract only
Barbour 2010[9]	Descriptive- Brain imaging study	Johnsen 2023[10]	Duplicate of Hemager 2018
Bauer 2019[11]	Cognition used as predictor of group status	Johnson 2012[12]	Wrong exposure
Bell 2023[13]	Wrong exposure	Kaplan 2011[14]	Wrong exposure
Biederman 2016[15]	Abstract only	Kauffman 1979[16]	Wrong outcome
Bilu 2025[17]	No relevant data on exposure-outcome link	Kavanaugh 2025[18]	Wrong exposure
Blain 2025[19]	Wrong population	Kenar 2013[20]	Abstract only
Bohon 2007[21]	No extractable data	Kestenbaum 1980[22]	Wrong outcome
Boisvert 2025[23]	No extractable data	Knudsen 2022[24]	Duplicate of Hemager 2018 and Andreassen 2024
Bora 2019[25]	No extractable data	Knudsen 2023[26]	Duplicate of Hemager 2018
Bornstein 2021[27]	Wrong exposure	Knudsen 2023[28]	Wrong study design
Boukhari 2019[29]	Abstract only	Lai 2002[30]	Wrong exposure
Boukhari 2019[31]	Abstract only	Landau 1972[32]	Wrong study design
Brennan 2000[33]	Wrong exposure	Le 2017[34]	Wrong exposure
Brunovsky 2020[35]	Abstract only	Lin 2015[36]	Duplicate of Lin K 2017
Burger 2022[37]	Wrong exposure	Liu 2022[38]	Wrong exposure
Burger 2023[39]	Duplicate of Burger 2022	Liu 2025[40]	Wrong outcome
Camprodon-Boadas 2021[41]	Abstract only	Marcus 1993[42]	Wrong outcome
Can 2018[43]	Abstract only	McCormack 2016[44]	Books or book chapters
Caplan 1989[45]	Wrong exposure	McDonough-Ryan 2001[46]	Duplicate of McDonough-Ryan 2002
Christiani 2013[47]	Abstract only	McNeil 1993[48]	Wrong outcome
Christiani 2014[49]	Abstract only	Meyer 2018[50]	Wrong outcome
Christiani 2014[51]	Abstract only	Misiak 2021[52]	Not relevant
Christiani 2019[53]	Wrong comparator	Mubasyiroh 2025[54]	Wrong exposure
Christiani 2022[55]	Duplicate of Hemager 2018	Murage 2025[56]	Wrong outcome
Curl 2025[57]	Conference abstract	Murphy 2018[58]	Abstract only
D'Angelo 1993[59]	No enough data to calculate SMD	Nayberg 2017[60]	Abstract only
Dave 2009[61]	Wrong outcome	Ng 2018[62]	Wrong exposure
De la Serna 2011[63]	Duplicate of De la Serna 2020	Ng 2025[64]	Wrong outcome
De la Serna 2016[65]	Duplicate of De la Serna 2020	Noniyeva 2018[66]	Abstract only
De la Serna 2017[67]	Duplicate of De la Serna 2020	Novak 2018[68]	Abstract only
De la Serna 2010[69]	Wrong exposure	Nulman 2012[70]	Wrong exposure
De la Serna 2010[71]	Wrong study design	Pagliaccio 2020[72]	Wrong outcome
De la Serna 2017[73]	Abstract only	Pariante 2023[74]	Abstract only
De la Serna 2019[75]	Abstract only	Parrilla-Escobar 2024[76]	Wrong exposure
De Zwarte 2017[77]	Wrong outcome	Patil 2025[78]	No healthy control group
De Zwarte 2018[79]	Abstract only	Peredo 2020[80]	Wrong outcome
De Zwarte 2018[81]	Duplicate of Haren 2018	Prasad 2009[82]	Wrong outcome
De Zwarte 2022[83]	Wrong comparator	Razaz 2016[84]	Wrong exposure

Dickson 2018[85]	Wrong exposure	Remberk 2011[86]	Wrong patient population
Diwadkar 2012[87]	Descriptive- Brain imaging study	Rogers 2023[88]	Wrong exposure
Diwadkar 2013[89]	Abstract only	Rolf 1972[90]	Wrong study design
Duffy 2009[91]	Wrong comparator	Ross 2008[92]	Wrong study design
Eriksen 2015[93]	Exposure not MDD (self-reported depression only)	Salisbury 2011[94]	Wrong outcome
Espie 2012[95]	Wrong outcome	Schrijver 2023[96]	Wrong exposure
Florsheim 1974[97]	Wrong study design	Schubert 2007[98]	Duplicate of Schubert 2005
Fricke 2025[99]	Wrong outcome	Seidman 2013[100]	Wrong exposure
Friedman 2000[101]	Wrong study design	Sharma 2011[102]	Conference abstract
Gilbert 2018[103]	Wrong outcome	Short 2013[104]	Abstract only
Gilmore 2017[105]	Abstract only	Sidorchuk 2023[106]	No specific parental SMI data
Goetz 2017[107]	Duplicate of Goetz 2019	Siegel-Ramsay 2025[108]	Conference abstract
Goetz 2018[109]	Abstract only	Sinha 2021[110]	Wrong exposure
Gregersen 2022[111]	Duplicate of Hemager 2018	Skurtveit 2014[112]	Wrong exposure
Greve 2024[113]	Duplicate of De la Serna 2020	Stephens 2021[114]	Abstract only
Greve 2022[115]	Duplicate of Hemager 2018	Stephens 2024[116]	Inability to convert data to SMD
Grunebaum 1978[117]	Wrong outcome	Sugranyes 2017[118]	Abstract only
Gumley 2017[119]	Abstract only	Sugranyes 2017[120]	Duplicate of Boadas 2023 and DelaSerna 2020
Gunnarsdottir 2018[121]	Wrong outcome	Sugranyes 2021[122]	Duplicate of De la Serna (2020) and the brain imaging study
Gustafsson 2018[123]	Wrong exposure	Thorup 2012[124]	Conference abstract
Hankin 2025[125]	Wrong outcome	Thorup 2018[126]	Conference abstract
Hans 1999[127]	Wrong outcome	Tognin 2025[128]	Wrong exposure
Hans 2005[129]	Wrong study design	Tsypes 2016[130]	Wrong exposure
Hans 2009[131]	Duplicate of Auerbach 2022	Ucok Demir 2015[132]	Abstract only
Harjan 1989[133]	Abstract only	Valli 2021[134]	Duplicate of Camprodon-Boadas 2023 and DelaSerna 2020
Hemager 2012[135]	Abstract only	VanHaren 2017[136]	Wrong outcome
Hemager 2013[137]	Abstract only	Ver Loren van Themaat 2020[138]	Duplicate of Hemager 2018
Hemager 2014[139]	Abstract only	Wisner 2015[140]	Abstract only
Hemager 2018[141]	Abstract only	Woody 2022[142]	Wrong outcome
Hemager 2019[143]	Duplicate of Hemager 2018	Xie 2025[144]	No extractable data
Hemager 2021[145]	Duplicate of Hemager 2018	Zahn-Waxler 1984[146]	Wrong outcome

eTable 3. Diagnostic methods for parental severe mental illness in included studies

Study	Severe mental illness type	Diagnosis or measurement
Anderson 1993[147]	Maternal MDD, BP	SADS-L
Andreassen 2024[148]	Parental schizophrenia, BP	ICD-10 or 8
Arıcı-Saglıyan 2025[149]	Parental BP	DSM-5
Asarnow 2014[150]	Maternal MDD	DSM-IV
Auerbach 2022[151]	Parental schizophrenia	SADS-L
Burger 2022[152]	Maternal schizophrenia, MDD	DSM-V
Burton 2018[153]	Parental schizophrenia, BP	ICD-10/8
Camprodon-Boadas 2023[154]	Parental schizophrenia, BP	Hospital/clinical data
Can 2019[155]	Parental BP	Clinical/hospital registry
Chai 2016[156]	Parental MDD	DSM-IV
Christiani 2019[157]	Parental schizophrenia, BP	ICD-10/8

Cicchetti 2000[158]	Maternal MDD	DSM-III-R
Conroy 2012[159]	Maternal MDD	DSM-IV
Davalos 2004[160]	Parental schizophrenia	DSM-IV
De la Serna 2020[161]	Parental schizophrenia, BP	DSM-IV
Deveci 2013[162]	Parental BP	DSM-IV
Diwadkar 2011[163]	Parental schizophrenia, BP	DSM-IV
ElSehrawy 2022[164]	Parental schizophrenia	DSM-IV
Fattahi 2015[165]	Maternal MDD	DSM-IV
Fis 2008[166]	Parental schizophrenia	DSM IV
Galbally 2011[167]	Maternal MDD	DSM-IV
Goetz 2019[168]	Parental BP	DSM-V
Goldstein 2000[169]	Parental schizophrenia	DSM-IV
Goodman 1987[170]	Maternal MDD, schizophrenia	DSM-III
Gotlib 2005[171]	Parental BP	SCID
Gumustas 2018[172]	Parental schizophrenia	DSM-IV
Hanford 2016[173]	Parental BP	DSM-IV
Hanley 2013[174]	Maternal MDD	DSM-IV
Haren 2018[175]	Parental schizophrenia, BP	DSM-IV
Hay 2001[176]	Maternal MDD	DSM-IV
Hemager 2018[177]	Parental schizophrenia, BP	ICD-10/8
Henriksson 2004[178]	Maternal schizophrenia	RDC
Hirose 1997[179]	Maternal MDD	DSM III
Horton 2017[180]	Parental schizophrenia	DSM-IV
HowesVallis 2020[181]	Parental MDD, BP, schizophrenia	DSM-5
Huang 2011[182]	Parental MDD	DSM-IV
Jundong 2012[183]	Parental schizophrenia	ICD-8/9/10
Kersten-Alvarez 2012[184]	Maternal MDD	DSM-IV
Kim 2017[185]	Parental BP	DSM (SCID)
Klimes-Dougan 2006[186]	Maternal MDD, BP	SADS-L
Kluczniok 2016[187]	Maternal MDD	DSM-IV
Kujawa 2014[188]	Parental MDD	DSM-IV
Levitan 2024[189]	Maternal MDD	PHQ
Lin 2024[190]	Parental MDD	ICD-9-CM
Lin 2025[191]	Parental MDD	ICD-9-CM
Lin A 2017[192]	Maternal schizophrenia, BP, MDD	ICD-9
Lin K 2017[193]	Parenta BP	DSM-IV
Lopez-Duran 2013[194]	Parental MDD	DSM-IV
MacKenzie 2020[195]	Parental SMI (schizophrenia, BP, MDD)	DSM-IV and DSM-5
Marothi 2014[196]	Maternal schizophrenia, BP	DSM-IV
Maselko 2015[197]	Maternal MDD	DSM-IV
Maziade 2009[198]	Parental schizophrenia, BP	DSM-III-R [SCID]
McDonough-Ryan 2002[199]	Parental BP	DSM-IV
Meiser 2015[200]	Maternal MDD	DSM-IV
Micco 2009[201]	Parental MDD	DSM-III-R
Milgrom 2004[202]	Maternal MDD	HDRS
Monk 2008[203]	Parental MDD	DSM-IV
Morgan 2012[204]	Maternal schizophrenia, BP, MDD	ICD-9
Murray 1996[205]	Maternal MDD	DSM III
Murray 2010[206]	Maternal MDD	DSM-IV
Nulman 2002[207]	Maternal MDD	DSM-IV
Oberlander 2007[208]	Maternal MDD	DSM-IV
O'Leary 2019[209]	Maternal MDD	HDRS
Oner 2005[210]	Parental schizophrenia	DSM-IV
Osborne 2022[211]	Maternal MDD	DSM-IV
Ozan 2010[212]	Parental schizophrenia	DSM-IV
Patino 2013[213]	Parental BP	DSM-IV
Perez-Edgar 2006[214]	Parental MDD	DSM-IV
Pine 2005[215]	Parental MDD	DSM-IV

Propper 2023[216]	Parental BP, MDD	DSM-IV and DSM-5
Quevedo 2012[217]	Maternal MDD	MINI
Raju 2025[218]	Maternal Postpartum Psychosis	ICD-10
Ranning 2018[219]	Parental schizophrenia, BP	ICD-8/10
Restrepo-Mejia 2023[220]	Parental BP	DSM-V
Rieder 1977[221]	Parental schizophrenia	Hospital records
Rybakowski 2009[222]	Parental BP	DSM-IV
Sanchez-Gutierrez 2020[223]	Parental schizophrenia	Hospital/clinical data
Santucci 2014[224]	Maternal MDD	DSM-IV
Santucci 2017[225]	Maternal BP	DSM-IV
Saxena 2023[226]	Parental BP	Clinical diagnosis
Schreiber 1992[227]	Parental schizophrenia	DSM-III
Schreiber 1997[228]	Parental schizophrenia	DSM-III
Schubert 2005[229]	Maternal schizophrenia, affective psychosis	Psychiatric records
Segura 2024[230]	Parental schizophrenia, BP	Hospital/clinical data
Sharma 2017[231]	Parental BP	DSM-IV
Simonetti 2021[232]	Parental BP	DSM-V
Singh 2018[233]	Parental BP, MDD	DSM–IV
Spang 2021[234]	Parental schizophrenia, BP	ICD-10/8
Sunew 2004[235]	Maternal MDD	DSM-III-R
Taylor 1999[236]	Maternal MDD	DSM III
Topal 2021[237]	Parental MDD, BP	DSM-IV
Traill 2002[238]	Maternal MDD	DSM-IV
Veddum 2022[239]	Parental schizophrenia, BP	ICD-10/8
Venezia 2021[240]	Parental MDD	DSM-IV
Ver Loren van Themaat 2021[241]	Parental schizophrenia, BP	ICD-10/8
Versace 2010[242]	Parental BP	DSM-IV
Watt 1982[243]	Parental schizophrenia	DSM-III
Weissman 1986[244]	Parental MDD	RDC
Welge 2016[245]	Parental BP	DSM-IV
Whiffen 1989[246]	Maternal MDD	DSM III
Whitney 2013[247]	Parental BP	DSM
Winters 1981[248]	Parental l MDD, BP, schizophrenia	DSM-II
Wolf 2002[249]	Parental schizophrenia, MDD	SADS-L
Woody 2015[250]	Maternal MDD	DSM-IV
Worland 1980[251]	Parental schizophrenia, BP	DSM-II
Yazkan-Akgul 2022[252]	Parental psychotic disorders	DSM-V
Yoshida 1999[253]	Maternal schizophrenia	RDC
Zhou 2017[254]	Parental schizophrenia	DSM-IV
Zhu 2023[255]	Parental BP	DSM-V

BP, Bipolar disorder; BRIEF, Behaviour Rating Inventory of Executive Function; DSM, Diagnostic and Statistical Manual of Mental Disorders; HDRS, Hamilton Depression Rating Scale; ICD, International Classification of Diseases; MINI, Mini International Neuropsychiatric Interview; MDD, Major depressive disorder; PHQ, Patient Health Questionnaire; RDC, Research Diagnostic Criteria; SADS-L, Schedule for Affective Disorders and Schizophrenia – Lifetime Version; SCID, Structured Clinical Interview for DSM.

eTable 4a. Specific cognitive measures and corresponding domains in studies of parental schizophrenia and offspring cognitive outcomes.

Study	Cognitive measure	Cognitive domain
Andreassen 2024[148]	Behavioral Regulation Index	Executive function
	Emotional Control	Executive function
	Emotional Regulation Index	Executive function
	General Executive Composite (GEC)	Executive function
	Inhibit	Executive function
	Initiate	Executive function
	Metacognition Index	Executive function
	Organization of Materials	Executive function
	Planning/Organization	Executive function

	Self Monitor	Executive function
	Shift	Executive function
	Task Monitor	Executive function
	Working Memory	Executive function
	Reynolds Intelligence Screening Test (RIST) index	IQ
Auerbach 2022[151]	Attention	Attention
	Trail Making Test B	Executive function
	Trail Making Test B Errors	Executive function
	Wisconsin Card Sorting Test (WCST) Categories	Executive function
	WCST Category Score	Executive function
	WCST Perseverative Errors	Executive function
	Wechsler Memory Test (WMS) faces (delayed)	Memory
	WMS faces (immediate)	Memory
	WMS logical memory 1 immediate	Memory
	WMS logical memory 2 delayed	Memory
	WMS visual reproduction 1 (immediate)	Memory
	WMS visual reproduction 2 (delayed)	Memory
Burger 2022[152]	Overall Cognition	General cognitive
	Bayley Scales of Infant Development-3rd edition (BSID-III) - Expressive	Language
	BSID-III Language Composite Score	Language
	BSID-III Receptive	Language
Burton 2018[153]	Conners' Continuous Performance Test, 2nd edition (CPT II) – Errors of commission	Attention
	Eriksen Flanker Task (EFT) – Accuracy (congruent trials)	Executive function
	EFT – Accuracy (incongruent trials)	Executive function
Camprodon-Boadas 2023[154]	Cognitive reserve	General cognitive
Christiani 2019[157]	Pragmatic Language (CCC-2 Total)	Language
	Pragmatic Language (General Communication Subscale)	Language
	Pragmatic Language (Social Interaction Subscale)	Language
	Receptive Language	Language
	Emotion Recognition (accuracy)	Social cognition
	Emotion Recognition (response latency)	Social cognition
	Ideational Generativity (Pattern Meanings)	Social cognition
	Theory of Mind – Animated Triangles (Accuracy)	Social cognition
	Theory of Mind – Animated Triangles (Intentionality)	Social cognition
	Theory of Mind – Strange Stories	Social cognition
	Theory of Mind – Strange Stories (response latency)	Social cognition
Davalos 2004[160]	Stopping Task	Attention
	Vocabulary	Language
	Word Knowledge	Language
	Working Memory - Counting Span	Memory
	Working Memoryv- Sentence Span	Memory
De la Serna 2020[161]	CPT Commissions	Attention
	CPT D'	Attention
	CPT Omissions	Attention
	CPT Perseverations	Attention
	CPT Reaction Time	Attention
	CPT Variability	Attention
	Stroop Interference	Executive function
	WCST Errors	Executive function
	WCST Perseverations	Executive function
	WCST Perseverative Errors	Executive function
	Global Ability Index	General cognitive
	Perceptual Reasoning	General cognitive
	Verbal Comprehension	Language

	Test of Memory and Learning (TOMAL) - Histories delayed recall	Memory
	TOMAL - Histories immediate recall	Memory
	TOMAL - Word List delayed recall	Memory
	TOMAL - Word List immediate recall	Memory
	WMS-III - delayed recall	Memory
	WMS-III - immediate recall	Memory
	Working Memory	Memory
	Processing speed	Processing speed
Diwadkar 2011[163]	Attention Memory	Attention
	Working Memory	Memory
Elsehrawy 2022[164]	Adaptability	Social cognition
	Affective disposition	Social cognition
	Emotion regulation	Social cognition
	Emotional expression	Social cognition
	Emotional perception	Social cognition
	Low impulsivity	Social cognition
	Peer relations	Social cognition
	Self-esteem	Social cognition
	Self-motivation	Social cognition
	Trait emotional intelligence scale child form (TEIQue-CF) - Total TEIQue score	Social cognition
Fis 2008[166]	Color Form Test	Executive function
	Progressive Figures Test	Executive function
	Trail Making Test B	Executive function
	WISC-R Block Design	General cognitive
	WISC-R Comprehension	General cognitive
	WISC-R Information	General cognitive
	WISC-R Object Assembly	General cognitive
	WISC-R Picture Completion	General cognitive
	Full-scale IQ	IQ
	Performance IQ	IQ
	Verbal IQ	IQ
	WISC-R Similarities	Language
	Trail Making Test A	Processing speed
	WISC coding	Processing speed
Goldstein 2000[169]	Full-scale IQ	IQ
Goodman 1987[170]	Full-scale IQ (IQMD)	IQ
Gumustas 2018[172]	Class repetition	Academic performance
	Reading difficulty	Academic performance
	School dropout	Academic performance
	Speech delay	Language
Haren 2018[175]	WISC-III - Full-scale IQ	IQ
	WAIS-III – Full-scale IQ	IQ
Hemager 2018[177]	RVP A′	Attention
	Intra-Extra Dimensional Set Shift	Executive function
	SOC PSIMM Problems Solved in Minimum Moves)	Executive function
	TMT Number-Letter Switching (Trail-Making Test)	Executive function
	Intelligence - Composite Score	General cognitive
	Intelligence - Guess What	General cognitive
	Intelligence - Odd-Item Out	General cognitive
	RIST Index	IQ estimate
	Verbal Fluency - Phonemic	Language
	Verbal Fluency - Semantic	Language
	Verbal Fluency - Switching	Language
	Memory for Stories (MFS) - Delayed Recall	Memory

	MFS - Immediate Recall	Memory
	RCFT - Immediate Recall	Memory
	Spatial Span Length	Memory
	SRM Percentage Correct	Memory
	SWM (Spatial Working Memory) Total Errors	Memory
	WISC-IV Arithmetic	Memory
	WISC-IV Letter-Number Sequencing	Memory
	Word Selective Reminding (WSR) - Delayed Recall	Memory
	WSR Immediate Recall	Memory
	TMT Letter Sequencing	Processing speed
	TMT Number Sequencing	Processing speed
	WISC Coding	Processing speed
	WISC Symbol Search	Processing speed
Henriksson 2004[178]	Language development	Language
Horton 2017[180]	Peabody Picture Vocabulary Test (PPVT-III)	IQ
	Emotion recognition – angry accuracy	Social cognition
	Emotion recognition – angry RT	Social cognition
	Emotion recognition – disgust accuracy	Social cognition
	Emotion recognition – disgust RT	Social cognition
	Emotion recognition – fear accuracy	Social cognition
	Emotion recognition – fear RT	Social cognition
	Emotion recognition – happy accuracy	Social cognition
	Emotion recognition – happy RT	Social cognition
	Emotion recognition – neutral accuracy	Social cognition
	Emotion recognition – neutral RT	Social cognition
	Emotion recognition – overall accuracy	Social cognition
	Emotion recognition – overall RT	Social cognition
	Emotion recognition – sad accuracy	Social cognition
	Emotion recognition – sad RT	Social cognition
HowesVallis 2020[181]	Visual memory performance (Rey Complex Figure Test [RCFT])	Memory
Jundong 2012[183]	School performance	Academic performance
Lin A 2017[192]	Numeracy	Academic performance
	Reading	Academic performance
	Spelling	Academic performance
	Writing	Academic performance
MacKenzie 2020[195]	RVP	Attention
	Intra-Extra Dimensional Set Shift	Executive function
	Letter-Number Sequencing	Executive function
	Matrix Reasoning	Executive function
	Stockings of Cambridge (CGT)	Executive function
	Stop Signal Task (SST)	Executive function
	Block Design	General cognitive
	Overall cognition (Wechsler Abbreviated Scale of Intelligence [WAS])	General cognitive
	WASI Similarities	Language
	WASI Verbal Fluency	Language
	WASI Vocabulary	Language
	Spatial Working Memory	Memory
	Verbal Memory - California Verbal Learning Test (CVLT)	Memory
	Verbal Memory - Story Memory	Memory
	Visual Memory - BVRT	Memory
	WISC Coding	Processing speed
Marothi 2014[196]	Full-scale IQ (WISC-IV)	IQ
	Modified Eyes Test	Social cognition
Maziade 2009[198]	CPT commissions	Attention
	CPT detectability d'	Attention

	CPT omissions	Attention
	CPT-hit reaction time block change	Attention
	CPT-hit standard error block change	Attention
	Stroop interference score	Attention
	Category fluency test	Executive function
	Letter fluency test	Executive function
	Total number of problems solved	Executive function
	Total rule violations	Executive function
	Total time violations	Executive function
	WCST failure to maintain set	Executive function
	WCST learning to learn	Executive function
	WCST number of categories completed	Executive function
	WCST total errors	Executive function
	WCST trials first category	Executive function
	Global IQ	IQ
	CVLT delayed recall:	Memory
	CVLT recognition	Memory
	CVLT total recall	Memory
	Rey delayed recall	Memory
	Rey immediate recall	Memory
	Rey recognition	Memory
	Total digit span	Memory
	Total spatial span	Memory
Morgan 2012[204]	Intellectual disability	General cognitive
Oner 2005[210]	Stroop – Interference	Executive function
	WCST – Categories completed	Executive function
	WCST – Percent correct	Executive function
	WCST – Perseverative responses	Executive function
	WCST – Total errors	Executive function
	Performance IQ	IQ
	Verbal IQ	IQ
Ozan 2010[212]	Auditory Consonant Trigram Test	Attention
	TOVA Comission errors	Attention
	TOVA Omission errors	Attention
	TOVA response time	Attention
	Stroop Test Main Card Reading Time	Executive function
	Trail Making Test B	Executive function
	WCST Category Score	Executive function
	WCST Total Correct Score	Executive function
	WCST Total Error Score	Executive function
	WCST Trials to Complete 1st Category	Executive function
	Controlled Word Association Test - Total Scores	Language
	Digit Span Test - Backwards Section Score	Memory
	Digit Span Test - Forwards Section Score	Memory
	Digit Span Test - Total Scores	Memory
	Rey Verbal Learning and Memory Test - Delayed Recalling Scores (7)	Memory
	Rey Verbal Learning and Memory Test - Recognition Percent Correct Score	Memory
	Rey Verbal Learning and Memory Test - Total Learning Scores (1–5)	Memory
	Rey Verbal Learning and Memory Test - True Positives	Memory
	Trail Making Test A	Processing speed
Raju 2025[218]	MacArthur–Bates Communicative Development Inventories (CDI) receptive vocabulary	Language
	Receptive–Expressive Emer-gent Language Test–Third Edition (REEL-3) - expressive language	Language
	REEL-3 receptive language	Language

Ranning 2018[219]	Education Register- High GPA (≥8)	Academic performance
	Education Register- Low GPA (<4)	Academic performance
	Education Register- No Graduation	Academic performance
Rieder 1977[221]	WISC - Full-scale IQ	IQ
Sanchez-Gutierrez 2020[223]	Commissions - Continuous Performance Test (CPT)	Attention
	D′ prime (CPT)	Attention
	Omissions (CPT)	Attention
	Perseverations (CPT)	Attention
	Reaction time (CPT)	Attention
	Variability (CPT)	Attention
	Categories (WCST)	Executive function
	Correct answers (WCST)	Executive function
	Errors (WCST)	Executive function
	Interference (Stroop)	Executive function
	Perseverations (WCST)	Executive function
	Perseverative errors (WCST)	Executive function
	Global IQ	IQ
	Perceptual Reasoning Index (PRI)	IQ
	Verbal Comprehension Index (VCI)	IQ
	Copy (Rey)	Memory
	Logical memory delayed recall (TOMAL test)	Memory
	Logical memory immediate recall (TOMAL test)	Memory
	Memory (Rey)	Memory
	Verbal learning delayed recall (TOMAL test)	Memory
	Verbal learning immediate recall (TOMAL test)	Memory
	Visual memory delayed recall (Wechsler Memory Scale II)	Memory
	Visual memory immediate recall (Wechsler Memory Scale II)	Memory
	Working Memory Index (WMI)	Memory
	Processing Speed Index	Processing speed
Schreiber 1992[227]	d2 Concentration Test	Attention
	d2 Total minus errors	Attention
	Mittenecker Perseveration Test	Executive function
	Wechsler Intelligence Scale (WIS)- Performance IQ	IQ
	WIS-Total IQ	IQ
	WIS- Verbal IQ	IQ
	Simple reaction time (auditory)	Processing speed
	Simple reaction time (visual)	Processing speed
Schreiber 1997[228]	School data - educational level score	Academic performance
	WIS - Full-scale IQ	IQ
Schubert 2005[229]	Selective attention test - compound hits	Attention
	Selective attention test - correct hits	Attention
	Selective attention test - Errors	Attention
	Grammatical Reasoning Test - Correct hits	Executive function
	Grammatical Reasoning Test - Correct rejections	Executive function
	Grammatical Reasoning Test - Errors	Executive function
	Grammatical Reasoning Test - Level of logical difficulty	Executive function
	Trail Making Test B	Executive function
	WCST Errors	Executive function
	WCST Perseverative responses	Executive function
	Block Design Test	General cognitive
	Verbal Fluency	Language
	Digit span test - Backward	Memory
	Digit span test - Forward	Memory
	Word-pair test - 1-hour delay	Memory
	Word-pair test - Immediate	Memory
	Reaction time	Processing speed

	Trail Making Test A, selective attention test	Processing speed
Segura 2024[230]	WISC-IV- General Ability Index (GAI)	General cognitive
Spang 2021[234]	Behavior Rating Inventory of Executive Function (BRIEF) Emotional Control	Executive function
	BRIEF Global Executive Composite (GEC)	Executive function
	BRIEF Inhibit	Executive function
	BRIEF Initiate	Executive function
	BRIEF Initiate:	Executive function
	BRIEF Monitor	Executive function
	BRIEF Organization of Materials	Executive function
	BRIEF Plan/Organize	Executive function
	BRIEF Shift	Executive function
	BRIEF Plan/Organize	Executive function
	BRIEF Working Memory	Memory
	BRIEF Working Memory:	Memory
Veddum 2022[239]	Random appropriateness	Social cognition
	Random intentionality	Social cognition
	Theory of Mind (ToM) appropriateness	Social cognition
	ToM intentionality	Social cognition
Ver Loren van Themaat 2021[241]	Attentional threshold (t0)	Attention
	Error rate	Attention
	Theory of visual - Visual STM (K)	Memory
	Processing speed (C)	Processing speed
Watt 1982[243]	Full-scale IQ	IQ
Winters 1981[248]	Distractibility task (digit span test)	Attention
	Non distractibility task (digit span test)	Attention
	WCST- Performance IQ	IQ
	WCST -Verbal IQ	IQ
	Word Communication task	Language
Wolf 2002[249]	Categories Completed (WCST)	Executive function
	Failure to Maintain Set ((WCST)	Executive function
	Perseverative Errors (WCST)	Executive function
	Perseverative Responses (WCST)	Executive function
	Total Errors (WCST)	Executive function
Worland 1980[251]	WISC - Full-scale IQ	IQ
Yazkan-Akgul 2022[252]	Controlled Oral Word Association Test (COWAT) - Total Word Number	Executive function
	Stroop Colour and Word Test (SCWT-5) - Total Duration	Executive function
	Trail Making Test B Total Duration	Executive function
	WCST Categories Completed	Executive function
	WCST Total Correct	Executive function
	Full-scale IQ	IQ
	Vocabulary	Language
	California Verbal Learning Test (CVLT) - Discriminality	Memory
	CVLT - Long Delay Recall	Memory
	CVLT - Recognition	Memory
	CVLT - Short Delay Recall	Memory
	Trail Making Test A	Processing speed
	Diagnostic Analysis of Nonverbal Accuracy-2 (DANVA-2) Adult – Total error	Social cognition
	DANVA-2 Child – Total error	Social cognition
Yoshida 1999[253]	Bayley Scales of Infant Development – Mental Development Index (BSID – MDI)	General cognitive
Zhou 2017[254]	Wisconsin_Categories Completed	Executive function
	Wisconsin_Nonperseverative Errors	Executive function
	Wisconsin_Perseverative Errors	Executive function
	Wisconsin_Total Correct	Executive function

	Wisconsin_Total Errors	Executive function

eTable 4b. Specific cognitive measures and corresponding domains in studies of parental bipolar disorder and offspring cognitive outcomes.

Study	Cognitive measure	Cognitive domain
Anderson 1993[147]	Parent/child/teacher assessment - academic performance	Academic performance
Andreassen 2024[148]	Behavioral Regulation Index	Executive function
	Emotional Control	Executive function
	Emotional Regulation Index	Executive function
	General Executive Composite (GEC)	Executive function
	Inhibit	Executive function
	Initiate	Executive function
	Metacognition Index	Executive function
	Organization of Materials	Executive function
	Planning/Organization	Executive function
	Self Monitor	Executive function
	Shift	Executive function
	Task Monitor	Executive function
	Working Memory	Executive function
	RIST index	IQ
Arıcı-Saglıyan 2025[149]	Cancellation Test 1 – Marked Targets	Attention
	Cancellation Test 1 – Missed Targets	Attention
	Cancellation Test 1 – Total Errors	Attention
	Cancellation Test 2 – False Targets	Attention
	Cancellation Test 2 – Marked Targets	Attention
	Cancellation Test 2 – Missed Targets	Attention
	Cancellation Test 2 – Total Errors	Attention
	Cancellation Test 3 – False Targets	Attention
	Cancellation Test 3 – Marked Targets	Attention
	Cancellation Test 3 – Missed Targets	Attention
	Cancellation Test 3 – Total Errors	Attention
	Cancellation Test 4 – False Targets	Attention
	Cancellation Test 4 – Marked Targets	Attention
	Cancellation Test 4 – Missed Targets	Attention
	Cancellation Test 4 – Total Errors	Attention
	Stroop Errors Section 2	Attention
	Stroop Errors Section 4	Attention
	Stroop Errors Section 5	Attention
	Stroop Corrections Section 1	Executive function
	Stroop Corrections Section 2	Executive function
	Stroop Corrections Section 3	Executive function
	Stroop Corrections Section 4	Executive function
	Stroop Corrections Section 5	Executive function
	Serial Digit Learning Test – Total Score	Memory
	Cancellation Test 1 – Duration	Processing speed
	Cancellation Test 2 – Duration	Processing speed
	Cancellation Test 3 – Duration	Processing speed
	Cancellation Test 4 – Duration	Processing speed
	Stroop Duration Section 1	Processing speed
	Stroop Duration Section 2	Processing speed
	Stroop Duration Section 3	Processing speed
	Stroop Duration Section 4	Processing speed
	Stroop Duration Section 5	Processing speed

Burton 2018[153]	CPT II – Errors of commission	Attention
	EFT – Accuracy (congruent trials)	Executive function
	EFT – Accuracy (incongruent trials)	Executive function
Camprodon-Boadas 2023[154]	Cognitive reserve (WISC-IV/WAIS-IV)	General cognitive
Can 2019[155]	Executive Function/Speed Factor	Executive function
	Fluency/Central Executive Factor	Executive function
	Letter Fluency	Executive function
	Semantic Fluency	Executive function
	Trail Making Test B	Executive function
	WCST Perseverations	Executive function
	Global Cognition	General cognitive
	Auditory Consonant Trigrams Test (ACTT)	Memory
	Verbal Memory - Long Delay Recall (List 7)	Memory
	Verbal Memory - Rey Verbal Learning and Memory Test (RAVLT) Learning (Total 1-5)	Memory
	Verbal Memory - Recognition	Memory
	Verbal Memory - VerMem Factor	Memory
	Visual Memory - VisMem Factor	Memory
	Visual Memory - Visual Reproduction Test Delay Recall	Memory
	Visual Memory - Visual Reproduction Test Immediate Recall	Memory
	Working Memory - Phonologic Loop Factor	Memory
	Working Memory - WAIS Digit Span Backwards	Memory
	Working Memory - WAIS Digit Span Forwards	Memory
	Trail Making Test A	Processing speed
	Digit Symbol (Digit Symbol Substitution Test [DSST])	Processing speed
Christiani 2019[157]	Pragmatic Language (CCC-2 Total)	Language
	Pragmatic Language (General Communication Subscale)	Language
	Pragmatic Language (Social Interaction Subscale)	Language
	Receptive Language	Language
	Emotion Recognition (accuracy)	Social cognition
	Emotion Recognition (response latency)	Social cognition
	Ideational Generativity (Pattern Meanings)	Social cognition
	Theory of Mind – Animated Triangles (Accuracy)	Social cognition
	Theory of Mind – Animated Triangles (Intentionality)	Social cognition
	Theory of Mind – Strange Stories	Social cognition
	Theory of Mind – Strange Stories (response latency)	Social cognition
De la Serna 2020[161]	CPT Commissions	Attention
	CPT D'	Attention
	CPT Omissions	Attention
	CPT Perseverations	Attention
	CPT Reaction Time	Attention
	CPT Variability	Attention
	Stroop Interference	Executive function
	WCST Errors	Executive function
	WCST Perseverations	Executive function
	WCST Perseverative Errors	Executive function
	Global Ability Index	General cognitive
	Perceptual Reasoning	General cognitive
	Verbal Comprehension	Language
	Logical Verbal Memory (TOMAL Histories DR)	Memory
	Logical Verbal Memory (TOMAL Histories IR)	Memory
	Visual Memory (WMS-III DR)	Memory
	Visual Memory (WMS-III IR)	Memory
	Working Memory	Memory
	Processing speed	Processing speed
Deveci 2013[162]	TOVA Test Scores-Comission errors	Attention

	TOVA Test Scores-Omission errors	Attention
	TOVA Test Scores-Response time	Attention
	Stroop Test - Main card reading time	Executive function
	Trail Making Test B	Executive function
	WCST Category score	Executive function
	WCST Total correct score	Executive function
	WCST Total error score	Executive function
	WCST Trials to complete first category	Executive function
	COWAT Total score	Language
	ACTT -Total scores	Memory
	Digit Span Test - Backward section score	Memory
	Digit Span Test - Forward section score	Memory
	Digit Span Test - Total scores	Memory
	Rey Verbal Learning and Memory Test (Delayed recalling scores)	Memory
	Rey Verbal Learning and Memory Test (Recognition percent correct score)	Memory
	Rey Verbal Learning and Memory Test (Total learning scores)	Memory
	Rey Verbal Learning and Memory Test (True positives)	Memory
	Trail Making Test A	Processing speed
Diwadkar 2011[163]	Sustained Attention	Attention
	Working Memory	Memory
Goetz 2019[168]	d2 Errors	Attention
	d2 Overall Performance	Attention
	d2 Sustained Attention	Attention
	Animal Sorting	Executive function
	Feature Identification False Alarms	Executive function
	Feature Identification RT	Executive function
	Shifting Set Commission Errors	Executive function
	Shifting Set Omission Errors	Executive function
	Shifting Set RT	Executive function
	Raven's Progressive Matrices	IQ
	Phonemic Fluency	Language
	Semantic Fluency	Language
	List Memory	Memory
	False Alarm Errors	Processing speed
	Reaction Time	Processing speed
	Affect Recognition	Social cognition
Gotlib 2005[171]	Emotion Stroop – Depressotypic	Executive function
	Emotion Stroop – Manic–Euphoric	Executive function
	Emotion Stroop – Manic–Irritable	Executive function
	Emotion Stroop – Neutral	Executive function
	Emotion Stroop – Physical Threat	Executive function
	Emotion Stroop – Social Threat	Executive function
	Negative adjectives endorsed	Memory
	Negative adjectives recalled	Memory
	Positive adjectives endorsed	Memory
	Positive adjectives recalled	Memory
Hanford 2016[173]	Full-scale IQ	IQ
Haren 2018[175]	Full-scale IQ	IQ
Hemager 2018[177]	Rapid Visual Information Processing A′ (RVP A′)	Attention
	IED (Intra-Extra Dimensional) Set Shift	Executive function
	SOC PSIMM (Stockings of Cambridge - Problems Solved in Minimum Moves)	Executive function
	TMT Number-Letter Switching (Trail-Making Test)	Executive function
	Intelligence - Composite Score	General cognitive
	Intelligence - Guess What	General cognitive

	Intelligence - Odd-Item Out	General cognitive
	RIST index	IQ
	Verbal Fluency - Phonemic	Language
	Verbal Fluency - Semantic	Language
	Verbal Fluency - Switching	Language
	MFS Delayed Recall	Memory
	MFS Immediate Recall	Memory
	RCFT Immediate Recall	Memory
	Spatial Span Length	Memory
	Spatial Working Memory - Total Errors	Memory
	Stockings of Cambridge (SRM) Percentage Correct	Memory
	WISC-IV Arithmetic	Memory
	WISC-IV Letter-Number Sequencing	Memory
	WSR Delayed Recall	Memory
	WSR Immediate Recall	Memory
	TMT Letter Sequencing	Processing speed
	TMT Number Sequencing	Processing speed
	WISC-IV Coding	Processing speed
	WISC-IV Symbol Search	Processing speed
Kim 2017[185]	Full-scale IQ	IQ
Klimes-Dougan 2006[186]	CPT Dyscontrol	Attention
	CPT Impulsivity	Attention
	CPT Inattention	Attention
	Failure to Maintain Set	Executive function
	Trail Making Test B	Executive function
	Trials to Complete Category	Executive function
	WCST Categories Completed	Executive function
	WCST Conceptual Level Responses	Executive function
	WCST Executive Function Factor Score	Executive function
	WCST Perseverative Errors No	Executive function
	WCST Perseverative Responses No	Executive function
	WCST Total Errors No	Executive function
	Full-scale IQ	IQ
	CVLT Long Free Recall	Memory
	CVLT Perseverations Trials 1 to 5	Memory
	CVLT Short Free Recall	Memory
	CVLT Trials 1 to 5 totaI	Memory
	Rey-Osterieth - Recall Organization	Memory
	Rey-Osterieth - Recall Total	Memory
	Trail Making Test A	Processing speed
Lin A 2017[192]	Numeracy	Academic performance
	Reading	Academic performance
	Spelling	Academic performance
	Writing	Academic performance
Lin K 2017[193]	Continuous Performance Task	Attention
	TOL Executive time	Executive function
	TOL Number of problems solved	Executive function
	TOL Total number of moves	Executive function
	TOL Total time	Executive function
	Tower of London - Total time	Executive function
	NAB-Mazes	Language
	Brief Visuospatial Memory Test-Revised	Memory
	Hopkins Verbal Learning Test-Revised	Memory
	WMS-III SS	Memory
	BACS-Symbol Coding	Processing speed
	Trail Making Test A	Processing speed
Marothi 2014[196]	Full-scale IQ	IQ
	The Eyes Test	Social cognition

Maziade 2009[198]	CPT commissions	Attention
	CPT D'	Attention
	CPT omissions	Attention
	CPT-hit reaction time block change	Attention
	CPT-hit standard error block change	Attention
	Selective attention - Stroop interference score	Attention
	Category fluency test	Executive function
	Letter fluency test	Executive function
	TOLDX Total number of problems solved in minimum moves	Executive function
	TOLDX Total rule violations	Executive function
	TOLDX Total time violations	Executive function
	WCST failure to maintain set	Executive function
	WCST learning to learn	Executive function
	WCST number of categories completed	Executive function
	WCST Total Errors	Executive function
	WCST trials first category	Executive function
	Full-scale IQ	IQ
	Verbal episodic memory - CVLT delayed recall	Memory
	Verbal episodic memory - CVLT recognition	Memory
	Verbal episodic memory - CVLT total recall	Memory
	Visual episodic memory - Rey delayed recall	Memory
	Visual episodic memory - Rey immediate recall	Memory
	Visual episodic memory - Rey recognition	Memory
	Working memory - Total digit span	Memory
	Working memory - Total spatial span	Memory
McDonough-Ryan 2002[199]	Arithmetic	Academic performance
	Reading	Academic performance
	Spelling	Academic performance
	Full-scale IQ	IQ
	Performance IQ	IQ
	Verbal IQ	IQ
Morgan 2012[204]	Intellectual disability	General cognitive
Patino 2013[213]	Intrasubject Variability Congruent	Executive function
	Intrasubject Variability Incongruent	Executive function
	Intrasubject Variability Neutral	Executive function
	Proportion Correct Congruent	Executive function
	Proportion Correct Incongruent	Executive function
	Proportion Correct Neutral	Executive function
	Response Time Congruent	Executive function
	Response Time Incongruent	Executive function
	Response Time Neutral	Executive function
	WASI-II Full-Scale IQ	IQ
Propper 2023[216]	Intellectual disability	General cognitive
	Learning disorder	General cognitive
Ranning 2018[219]	High GPA (≥8)	Academic performance
	Low GPA (<4)	Academic performance
	No Graduation	Academic performance
Restrepo-Mejia 2023[220]	Auditory attention (ENI)	Attention
	Auditory Performance	Attention
	Visual performance correct answers	Attention
	Phonological fluency A	Executive function
	Phonological fluency F	Executive function
	Phonological fluency F-A-S	Executive function
	Phonological fluency S	Executive function
	Semantic fluency	Executive function
	STROOP conflict	Executive function
	Trail Making Test B	Executive function

	WCST Categories	Executive function
	WCST Errors	Executive function
	WCST Perseverative Errors	Executive function
	Intellectual Ability	General cognitive
	Rey Figure Copy	General cognitive
	Rey Figure Time Copy	General cognitive
	Rey figure evocation	Memory
	Rey figure evocation time	Memory
	Story recall	Memory
	Story retrieval	Memory
	STROOP Colour	Processing speed
	STROOP Word	Processing speed
	Trail Making Test A	Processing speed
	Visual Performance time	Processing speed
Rybakowski 2009[222]	WCST-%conc (Conceptual Responses %)	Executive function
	WCST-CC (Categories Completed)	Executive function
	WCSTh-1st CAT (Trials to First Category)	Executive function
	WCST-NP (Non-Perseverative Errors)	Executive function
	WCST-P (Perseverative Errors)	Executive function
Santucci 2017[225]	BSID - MDI	General cognitive
Saxena 2023[226]	AGN CE-negative (n)	Executive function
	AGN CE-positive (n)	Executive function
	AGN Omissions-negative (n)	Executive function
	AGN Omissions-positive (n)	Executive function
	AGN RT-negative (sec.)	Executive function
	AGN RT-positive (sec.)	Executive function
	CGT Delay aversion	Executive function
	CGT Deliberation time (msec.)	Executive function
	CGT Proportion bet	Executive function
	CGT Quality of decision making	Executive function
	CGT Risk adjustment	Executive function
	CGT Risk taking	Executive function
	SOC Moves (n)	Executive function
	Big/Little Circle (BLC) % correct selection	General cognitive
	Match to Sample (MTS) visual search S % correct choice	General cognitive
	WASI-II Full-Scale IQ	IQ
Segura 2024[230]	General Ability Index (GAI)	General cognitive
Sharma 2017[231]	Full-scale IQ	IQ
	Performance IQ	IQ
	Verbal IQ	IQ
Simonetti 2021[232]	Spatial Recognition Memory – % Correct	Memory
Singh 2018[233]	Number–Letter Switching	Executive function
	Full-scale IQ	IQ
	Letter Sequencing	Processing speed
	Motor Speed	Processing speed
	Number Sequencing	Processing speed
	Visual Scanning	Processing speed
Spang 2021[234]	Behavior Rating Inventory of Executive Function (BRIEF) Emotional Control	Executive function
	BRIEF Global Executive Composite	Executive function
	BRIEF Inhibit	Executive function
	BRIEF Initiate	Executive function
	BRIEF Monitor	Executive function
	BRIEF Organization of Materials	Executive function
	BRIEF Plan/Organize	Executive function
	BRIEF Shift	Executive function

	BRIEF Working Memory	Memory
Topal 2021[237]	Correct responses, Emotional Word-Face Stroop, Block II	Executive function
	Correct responses, Emotional Word-Face Stroop, Block III	Executive function
	Correct responses, Emotional Word-Face Stroop, Block IV	Executive function
	Response latency (ms), Emotional Word-Face Stroop, Block II	Executive function
	Response latency (ms), Emotional Word-Face Stroop, Block III	Executive function
	Response latency (ms), Emotional Word-Face Stroop, Block IV	Executive function
Veddum 2022[239]	Random appropriateness	Social cognition
	Random intentionality	Social cognition
	Theory of Mind (ToM) appropriateness	Social cognition
	ToM intentionality	Social cognition
Ver Loren van Themaat 2021[241]	Motor Screening – Errors (theory of visual attention [TVA])	Attention
	Threshold of visual conscious perception (t0) (TVA)	Attention
	Visual attention error rate (TVA)	Attention
	Visual short-term memory capacity (K) (TVA)	Memory
	Motor Screening – Latency (TVA)	Processing speed
	Visual processing speed (C; digits/second) (TVA)	Processing speed
Versace 2010[242]	WASI - Full-scale IQ	IQ
Welge 2016[245]	WASI - Full-scale IQ	IQ
Whitney 2013[247]	WASI - Full-scale IQ	IQ
	Diagnostic test of nonverbal accuracy 2 (DANVA) – Adult Faces	Social cognition
	DANVA – Child Faces	Social cognition
	NEPSY Affect Recognition	Social cognition
	NEPSY Theory of Mind	Social cognition
	Social Responsiveness Scale (SRS) – Total	Social cognition
Winters 1981[248]	Distractibility task (digit span test)	Attention
	Non distractibility task (digit span test)	Attention
	WISC- Performance IQ	IQ
	WISC- Verbal IQ	IQ
	Word Communication task	Language
Worland 1980[251]	Full-scale IQ	IQ
Zhu 2023[255]	Affective Go/No-go response time to negative stimuli	Executive function
	Affective Go/No-go response time to positive stimuli	Executive function
	WASI-II Full-Scale IQ	IQ

eTable 4c. Specific cognitive measures and corresponding domains in studies of parental major depressive disorder and offspring cognitive outcomes

Study	Cognitive measure	Cognitive domain
Anderson 1993[147]	Parent, child and teacher assessment	Academic performance
Asarnow 2014[150]	WISC-IV Vocabulary (Met/Met)	Language
	WISC-IV Vocabulary (Val/Met)	Language
	WISC-IV Vocabulary (Val/Val)	Language
Burger 2022[152]	BSID - MDI	General cognitive
	BSID - Developmental Speech or Language Disorder	Language
	BSID - Expressive language	Language
	BSID - Receptive language	Language

Chai 2016[156]	Kaufman Brief Intelligence Test-2 (KBIT-2) - Full-scale IQ	IQ
Cicchetti 2000[158]	BSID - MDI	General cognitive
Conroy 2012[159]	BSID - MDI	General cognitive
Fattahi 2015[165]	Word Recall – Happy Stimuli	Memory
	Word Recall – Neutral Stimuli	Memory
	Word Recall – Sad Stimuli	Memory
Galbally 2011[167]	BSID - MDI	General cognitive
	Bayley Expressive Language	Language
	Bayley Receptive Language	Language
Goodman 1987[170]	IQ (IQMD) from BSID & McCarthy Scales	IQ
Hanley 2013[174]	BSID - MDI	General cognitive
	BSID-III Expressive Language	Language
	BSID-III Receptive Language	Language
Hay 2001[176]	WISC-III Full-scale IQ	IQ
Hirose 1997[179]	BSID - MDI	General cognitive
Huang 2011[182]	WISC - Full-scale IQ	IQ
Kersten-Alvarez 2012[184]	PPVT-R Verbal Intelligence	Language
Klimes-Dougan 2006[186]	CPT Dyscontrol	Attention
	CPT Impulsivity	Attention
	CPT Inattention	Attention
	Executive Function Factor Score	Executive function
	TMT Part B (Cognitive Flexibility & Set-Shifting)	Executive function
	WCST Categories Completed	Executive function
	WCST Categories Completed No	Executive function
	WCST Conceptual Level Responses	Executive function
	WCST Failure to Maintain Set	Executive function
	WCST Perseverative Errors No	Executive function
	WCST Perseverative Responses No	Executive function
	WCST Total Errors No	Executive function
	WCST Trials to Complete Category	Executive function
	Full-scale IQ	IQ
	CVLT Long Free Recall	Memory
	CVLT Perseverations Trials 1 to 5	Memory
	CVLT Short Free Recall	Memory
	CVLT Trials 1 to 5 total	Memory
	Rey-Osterieth - Recall Total	Memory
	Rey-Osterieth -Recall Organization	Memory
	Trail Making Test A	Processing speed
Kluczniok 2016[187]	Culture Fair Intelligence Test (IQ)	IQ
Kujawa 2014[188]	PPVT Verbal IQ	Language
Levitan 2024[189]	Flanker test score	Attention
	Dimensional Change Card Sort (cognitive flexibility)	Executive function
Lin 2024[190]	Intellectual Disability	General cognitive
	Developmental Speech or Language Disorder	Language
Lin 2025[191]	Intellectual disability	General cognitive
	Developmental speech/language disorder	Language
Lin A 2017[192]	Numeracy	Academic performance
	Reading	Academic performance
	Spelling	Academic performance
	Writing	Academic performance
Lopez-Duran 2013[194]	Emotion Discrimination (Sadness vs Anger)	Social cognition
	Emotion Recognition (Anger)	Social cognition
	Emotion Recognition (Sadness)	Social cognition
Maselko 2015[197]	Wechsler Preschool and Primary Scales of Intelligence (WPPSI) Visual-Spatial	General cognitive
	WPPSI Full-scale IQ	IQ

	WPPSI Verbal Comprehension	Language
Meiser 2015[200]	Reading the Mind in the Eyes Test-Emotion Labelling	Social cognition
	Reading the Mind in the Eyes Test-Emotion Recognition	Social cognition
Micco 2009[201]	Seidman CPT-Memory - False Alarms	Attention
	Seidman CPT-Memory - Hits	Attention
	Seidman CPT-Memory - Lates	Attention
	Seidman CPT-Memory - Omissions	Attention
	WISC-III Freedom from Distractibility (FDIQ)	Attention
	WCST Failure to Maintain Set	Executive function
	WCST Non-Perseverative Errors	Executive function
	WCST Perseverative Errors	Executive function
	WISC-III - Arithmetic	General cognitive
	WISC-III - Block Design	General cognitive
	Verbal Episodic Memory - CVLT-C T-score	Memory
	Working Memory - Digit Span	Memory
	Stroop Color-Word Score	Processing speed
	Stroop Interference Score	Processing speed
	Stroop Word Score	Processing speed
	WISC-III Digit-Symbol	Processing speed
	WISC-III Processing Speed Index (PSIQ)	Processing speed
	WISC-III Symbol Search	Processing speed
Milgrom 2004[202]	Early Screening Profiles (ESP) - Cognitive profile	General cognitive
	Early Screening Profiles (ESP) - Logical Relations subscale	General cognitive
	Early Screening Profiles (ESP) - Visual subscale	General cognitive
	WPPSI-R - Arithmetic	General cognitive
	WPPSI-R - Geometric design	General cognitive
	Full-scale IQ	IQ
	Performance IQ	IQ
	Verbal IQ	IQ
	Early Screening Profiles (ESP) - Basic Skills subscale	Language
	Early Screening Profiles (ESP) - Overall Language Profile	Language
	Early Screening Profiles (ESP) - Verbal subscale	Language
	WPPSI-R - Expressivity	Language
	WPPSI-R - Receptivity	Language
Monk 2008[203]	FSIQ	IQ
Morgan 2012[204]	Intellectual disability	General cognitive
Murray 1996[205]	General Cognitive Index (GCI)	General cognitive
	Verbal (McCarthy)	Language
	Memory (McCarthy)	Memory
Murray 2010[206]	General Certificate of Secondary Education (GCSE) - Academic Performance	Academic performance
	BSID - MDI	General cognitive
	McCarthy General Cognitive Ability	General cognitive
	WISC-III – Full-scale IQ	IQ
Nulman 2002[207]	BSID - MDI	General cognitive
	Global cognitive index (McCarthy Scale)	General cognitive
	Expressive language (Reynell Language Scales)	Language
	Verbal comprehension (Reynell Language Scales)	Language
Oberlander 2007[208]	BSID - MDI	General cognitive
	WPPSI language & visual-perceptual composite	IQ
O'Leary 2019[209]	BSID - MDI	General cognitive
	BSID - language development	Language
Osborne 2022[211]	BSID - MDI	General cognitive

	BSID - language development	Language
Perez-Edgar 2006[214]	Affective Posner – Invalid trials	Attention
	Affective Posner – Neutral trials	Attention
	Affective Posner – Valid trials	Attention
	Traditional Posner – Invalid trials	Attention
	Traditional Posner – Neutral trials	Attention
	Traditional Posner – Valid trials	Attention
Pine 2005[215]	Kaufman Brief Intelligence Test (KBIT) - Full Scale IQ	IQ
	Benton Test - Face recognition accuracy	Social cognition
Propper 2023[216]	Intellectual disability (Wexler Abbreviated Scale of Intelligence)	General cognitive
	Learning disorder (Wexler Abbreviated Scale of Intelligence)	General cognitive
Quevedo 2012[217]	BSID -Language	Language
Santucci 2014[224]	BSID - MDI	General cognitive
Singh 2018[233]	Number–Letter Switching	Executive function
	WASI- Full-scale IQ	IQ
	Delis–Kaplan Executive Function System (DKEFS) Letter Sequencing	Processing speed
	DKEFS Motor Speed	Processing speed
	DKEFS Number Sequencing	Processing speed
	DKEFS Visual Scanning	Processing speed
Sunew 2004[235]	Wechsler Individual Achievement Test (WIAT)-Composite	Academic performance
	WIAT-Mathematics	Academic performance
	WIAT-Reading	Academic performance
	WIAT-Spelling	Academic performance
	WISC-III-Block Design	General cognitive
	WISC-III-Picture Completion	General cognitive
	WISC-III-Estimated overall IQ	IQ
	WISC-III-Vocabulary	Language
	Diagnostic Analysis of Nonverbal Accuracy-2 (DANVA-2) - Adult Faces errors	Social cognition
	DANVA-2 - Child Faces errors	Social cognition
	Kusche Affective Interview – Revised (KAI-R) - Feelings vocabulary (negative words)	Social cognition
	KAI-R - Feelings vocabulary (positive words)	Social cognition
Taylor 1999[236]	PPVT percentile	Language
Topal 2021[237]	Correct responses, Emotional Word-Face Stroop, Block II	Executive function
	Correct responses, Emotional Word-Face Stroop, Block III	Executive function
	Correct responses, Emotional Word-Face Stroop, Block IV	Executive function
	Response latency (ms), Emotional Word-Face Stroop, Block II	Executive function
	Response latency (ms), Emotional Word-Face Stroop, Block III	Executive function
	Response latency (ms), Emotional Word-Face Stroop, Block IV	Executive function
Traill 2002[238]	Probe match – Angry face	Attention
	Probe match – Happy face	Attention
	Probe match – Sad face	Attention
	Probe mismatch – Angry face	Attention
	Probe mismatch – Happy face	Attention
	Probe mismatch – Sad face	Attention
	Total errors – Depressive list	Attention

	Total errors – Neutral list	Attention
	Total errors – Physical-threat list	Attention
	Total errors – Social-threat list	Attention
	Reading times – Depressive list	Executive function
	Reading times – Neutral list	Executive function
	Reading times – Physical-threat list	Executive function
	Reading times – Social-threat list	Executive function
	Endorsement – Negative words	Memory
	Endorsement – Positive words	Memory
	Recall – Negative words	Memory
	Recall – Positive words	Memory
Venezia 2021[240]	Attention Composite	Attention
	CPT	Attention
	Stroop Interference	Attention
	Go No-Go Task	Executive function
	Impulse Control Composite	Executive function
	Time Production	Executive function
	A, Not B Task	Memory
	Benton Visual Retention Test (VRT)	Memory
	N-Back Task	Memory
	Working Memory Composite	Memory
Weissman 1986[244]	WISC-R - Block Design	General cognitive
	PPVT	Language
	WISC - Vocabulary	Language
Whiffen 1989[246]	BSID - MDI	General cognitive
Winters 1981[248]	Distractibility task (digit span test)	Attention
	Non distractibility task (digit span test)	Attention
	WISC- Performance IQ	IQ
	WISC- Verbal IQ	IQ
	Word Communication	Language
Wolf 2002[249]	Categories Completed (WCST)	Executive function
	Failure to Maintain Set ((WCST)	Executive function
	Perseverative Errors (WCST)	Executive function
	Perseverative Responses (WCST)	Executive function
	Total Errors (WCST)	Executive function
Woody 2015[250]	Specific negative memories (Autobiographical Memory Test)	Memory
	Specific positive memories (Autobiographical Memory Test)	Memory
Yoshida 1999[253]	BSID - MDI	General cognitive

eTable 5. Summary of cognitive measure (subdomains) included in the general cognition domain

Cognitive measure	No of Studies	No of Estimates
BSID - MDI	15	38
Intellectual disability	4	9
Block Design Test	1	4
General Ability Index	1	4
Global Ability Index	1	4
Global cognitive index (McCarthy Scale)	3	4
Perceptual Reasoning	1	4
Cognitive reserve	1	2
Global cognition	1	2
Intelligence - Composite Score	1	2
Intelligence - Guess What	1	2
Intelligence - Odd-Item Out	1	2
Learning disorder	1	2
Overall cognition	2	2

WISC-III - Arithmetic	1	2
WISC-III - Block Design	1	2
WISC-III-Block Design	1	2
WISC-III-Picture Completion	1	2
Big/Little Circle % correct selection	1	1
Block Design	1	1
Early Screening Profiles - Cognitive profile	1	1
Early Screening Profiles - Logical Relations subscale	1	1
Early Screening Profiles - Visual subscale	1	1
Intellectual Ability	1	1
Match to Sample (MTS) visual search % correct choice	1	1
Rey Figure Copy	1	1
Rey Figure Time Copy	1	1
WISC-R - Block Design	1	1
WISC-R Block Design	1	1
WISC-R Comprehension	1	1
WISC-R Information	1	1
WISC-R Object Assembly	1	1
WISC-R Picture Completion	1	1
WPPSI Visual-Spatial	1	1
WPPSI-R - Arithmetic	1	1
WPPSI-R - Geometric design	1	1

Abbreviations: BSID–MDI: Bayley Scales of Infant Development – Mental Development Index; ESP: Early Screening Profiles; MTS: Match to Sample; WISC: Wechsler Intelligence Scale for Children; WISC-R: Wechsler Intelligence Scale for Children – Revised; WISC-III: Wechsler Intelligence Scale for Children – Third Edition; WPPSI: Wechsler Preschool and Primary Scale of Intelligence; WPPSI-R: Wechsler Preschool and Primary Scale of Intelligence – Revised.

eTable 6. Meta-regression of study-level characteristics and cognitive domains on offspring cognitive performance

Covariate	Schizophrenia (τ^2 = 0.098)<br>β (95% CI), p-value	Bipolar disorder (τ^2 = 0.219)<br>β (95% CI), p-value	Major depressive disorder (τ^2 = 0.069)<br>β (95% CI), p-value
Exposure type			
Maternal	0.19 (−0.10, 0.48), p=0.151	0.04 (−0.24, 0.32), p=0.730	−0.08 (−0.29, 0.13), p=0.443
Age group			
Childhood (6–12 years)	0.52 (−0.07, 1.11), p=0.076	−0.27 (−0.93, 0.38), p=0.253	0.02 (−0.20, 0.24), p=0.843
Adolescence (13–19 years)	0.49 (−0.18, 1.16), p=0.134	−0.41 (−1.11, 0.29), p=0.156	−0.05 (−0.34, 0.23), p=0.706
Adulthood (20+ years)	0.32 (−0.27, 0.92), p=0.252	−0.73 (−1.36, −0.10), p=0.030	−0.12 (−0.44, 0.20), p=0.420
Study quality			
Low	−0.63 (−1.05, −0.21), p=0.008	0.01 (−0.33, 0.34), p=0.963	−0.26 (−0.54, 0.02), p=0.065
Moderate	−0.24 (−0.45, −0.03), p=0.025	−0.12 (−0.34, 0.10), p=0.280	−0.09 (−0.28, 0.10), p=0.317
Publication year (centred)	−0.01 (−0.02, −0.00), p=0.040	0.00 (−0.01, 0.01), p=0.928	−0.00 (−0.01, 0.01), p=0.601
Cognitive domain			
Academic performance	0.40 (0.10, 0.71), p=0.014	0.10 (−0.34, 0.53), p=0.619	−0.25 (−1.32, 0.83), p=0.568
Attention	0.52 (0.19, 0.85), p=0.005	0.20 (−0.20, 0.60), p=0.301	0.06 (−0.53, 0.65), p=0.822
Executive function	0.39 (0.09, 0.68), p=0.013	0.04 (−0.29, 0.37), p=0.801	0.09 (−0.21, 0.39), p=0.514
General cognition	−0.12 (−0.36, 0.11), p=0.275	−0.14 (−0.66, 0.38), p=0.552	−0.02 (−0.33, 0.29), p=0.886
Language	0.37 (0.11, 0.64), p=0.009	0.08 (−0.25, 0.41), p=0.564	0.12 (−0.16, 0.40), p=0.374
Memory	0.32 (−0.10, 0.73), p=0.126	−0.07 (−0.42, 0.27), p=0.669	0.07 (−0.21, 0.36), p=0.573
Processing speed	0.06 (−0.36, 0.49), p=0.742	0.04 (−0.41, 0.49), p=0.835	−0.13 (−1.48, 1.22), p=0.695
Social cognition	0.51 (0.16, 0.86), p=0.008	0.19 (−0.15, 0.53), p=0.221	0.04 (−0.40, 0.48), p=0.820
No. studies	45	44	51

Reference categories were parental, early childhood (≤5 years), high study quality (NOS 8–10) and IQ. β coefficients represent differences in standardised mean differences relative to the reference category within each SMI model.

eTable 7. Effect sizes of cognitive outcomes in children of parents with severe mental illness, excluding large studies

Cognitive domain	No of Exposed	No of Controls	SMD (95% CI)	P-value	τ^2
Schizophrenia					
Academic performance	56	51	-0.33 (-7.56, 6.90)	0.666	0.63
Attention	943	1217	-0.33 (-0.59, -0.07)	0.015	0.23
Executive function	1248	1622	-0.34 (-0.52, -0.17)	0.001	0.12
IQ	1134	1320	-0.53 (-0.72, -0.34)	0.000	0.09
Language	974	1149	-0.70 (-1.20, -0.20)	0.010	0.36
Memory	809	1102	-0.58 (-1.08, -0.09)	0.024	0.44
Processing speed	530	793	-1.00 (-2.07, 0.07)	0.064	0.87
General cognitive	1037	3910	-1.07 (-1.92, -0.22)	0.019	0.49
Social cognition	493	513	-0.30 (-0.61, 0.01)	0.055	0.06
Overall	*3321*	*6362*	*-0.51 (-0.66, -0.37)*	*0.000*	*0.16*
Bipolar disorder					
Academic performance	46	62	-0.50 (-3.58, 2.58)	0.289	0.08
Attention	738	1037	-0.11 (-0.31, 0.09)	0.242	0.18
Executive function	1063	1434	-0.34 (-0.51, -0.16)	0.001	0.35
IQ	876	1100	-0.32 (-0.48, -0.17)	0.000	0.05
Language	543	765	-0.18 (-0.34, -0.02)	0.034	0.12
Memory	738	1009	-0.40 (-0.60, -0.19)	0.001	0.37
Processing speed	573	711	-0.57 (-1.42, 0.28)	0.165	1.18
General cognitive	1966	4018	-0.45 (-0.79, -0.12)	0.013	0.20
Social cognition	327	464	-0.02 (-0.26, 0.22)	0.818	0.05
Overall	*3423*	*6039*	*-0.30 (-0.39, -0.21)*	*0.000*	*0.15*
Major depressive disorder					
Academic performance	102	136	-0.60 (-2.02, 0.81)	0.208	0.27
Attention	447	569	-0.18 (-0.65, 0.28)	0.374	0.17
Executive function	501	597	-0.20 (-0.39, -0.01)	0.045	0.02
IQ	1113	892	-0.23 (-0.37, -0.08)	0.006	0.02
Language	8975	77567	-0.13 (-0.24, -0.02)	0.022	0.05
Memory	525	373	-0.20 (-0.41, 0.00)	0.052	0.19
Processing speed	163	114	-0.25 (-0.90, 0.41)	0.247	0.06
General cognitive	10360	80262	-0.23 (-0.34, -0.12)	0.000	0.20
Social cognition	179	150	-0.15 (-0.63, 0.33)	0.383	0.11
Overall	*11627*	*82247*	*-0.23 (-0.31, -0.15)*	*0.000*	*0.17*

CI: confidence interval, IQ: intelligence quotient, SMD: standardized mean difference

eTable 8. Effect sizes of cognitive outcomes in children of parents with severe mental illness, excluding low-quality studies as determined by the Newcastle-Ottawa Scale

Cognitive domain	No of Exposed	No of Controls	SMD (95% CI)	P-value	τ^2
Schizophrenia					
Academic performance	5814	1394701	-0.09 (-0.32, 0.13)	0.256	0.033
Attention	745	1032	-0.34 (-0.61, -0.07)	0.018	0.229
Executive function	1218	1592	-0.32 (-0.50, -0.14)	0.002	0.124
IQ	929	1127	-0.54 (-0.76, -0.32)	0.000	0.100
Language	697	869	-0.57 (-1.36, 0.22)	0.132	0.452
Memory	758	1051	-0.59 (-1.13, -0.05)	0.034	0.477
Processing speed	500	763	-1.06 (-2.28, 0.17)	0.081	0.951
Social cognition	465	484	-0.20 (-0.44, 0.04)	0.075	0.029
General cognitive	993	3845	-0.86 (-1.77, 0.05)	0.060	0.408
Overall	8809	1400733	-0.42 (-0.55, -0.29)	0.000	0.075
Bipolar disorder					
Academic performance	4618	764962	-0.16 (-0.55, 0.23)	0.218	0.026
Attention	650	903	-0.12 (-0.34, 0.10)	0.241	0.207

Executive function	959	1334	-0.28 (-0.43, -0.12)	0.001	0.267
IQ	687	866	-0.36 (-0.56, -0.16)	0.002	0.074
Language	455	631	-0.17 (-0.36, 0.03)	0.081	0.148
Memory	684	959	-0.38 (-0.61, -0.16)	0.002	0.388
Processing speed	519	661	-0.58 (-1.54, 0.37)	0.200	1.294
Social cognition	280	408	0.02 (-0.30, 0.35)	0.745	0.006
General cognitive	1912	3968	-0.39 (-0.74, -0.05)	0.030	0.202
Overall	7702	770605	-0.27 (-0.36, -0.18)	0.000	0.084
Major depressive disorder					
Academic performance	1862	88489	-0.46 (-1.25, 0.33)	0.161	0.203
Attention	355	418	-0.22 (-0.85, 0.42)	0.395	0.159
Executive function	501	597	-0.20 (-0.39, -0.01)	0.045	0.024
IQ	1037	758	-0.20 (-0.35, -0.05)	0.014	0.015
Language	16461	153328	-0.09 (-0.19, 0.01)	0.064	0.031
Memory	497	344	-0.20 (-0.45, 0.05)	0.094	0.041
Processing speed	163	114	-0.25 (-0.90, 0.41)	0.247	0.056
Social cognition	179	150	-0.15 (-0.63, 0.33)	0.383	0.106
General cognitive	17922	156157	-0.22 (-0.33, -0.10)	0.001	0.082
Overall	20829	246315	-0.21 (-0.29, -0.13)	0.000	0.060

CI: confidence interval, IQ: intelligence quotient, SMD: standardized mean difference

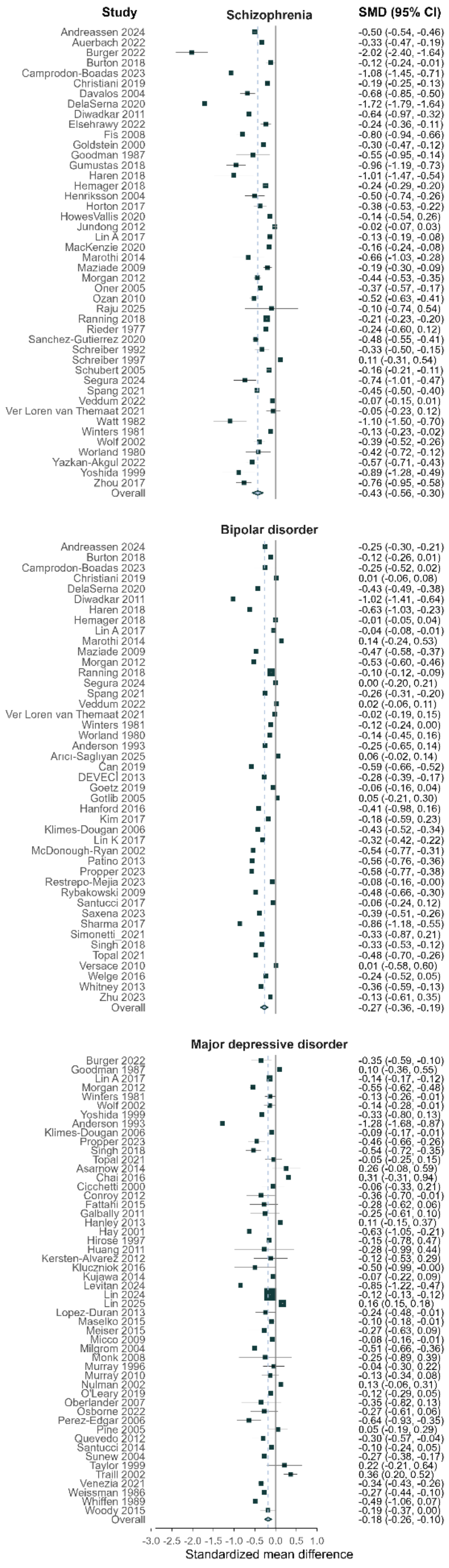

τ^2 (schizophrenia = 0.081; bipolar disorder = 0.083, major depressive disorder = 0.035)

eFigure 1. Forest plot of the association between parental severe mental illness and offspring overall cognitive outcomes after exclusion of the general cognition domain.

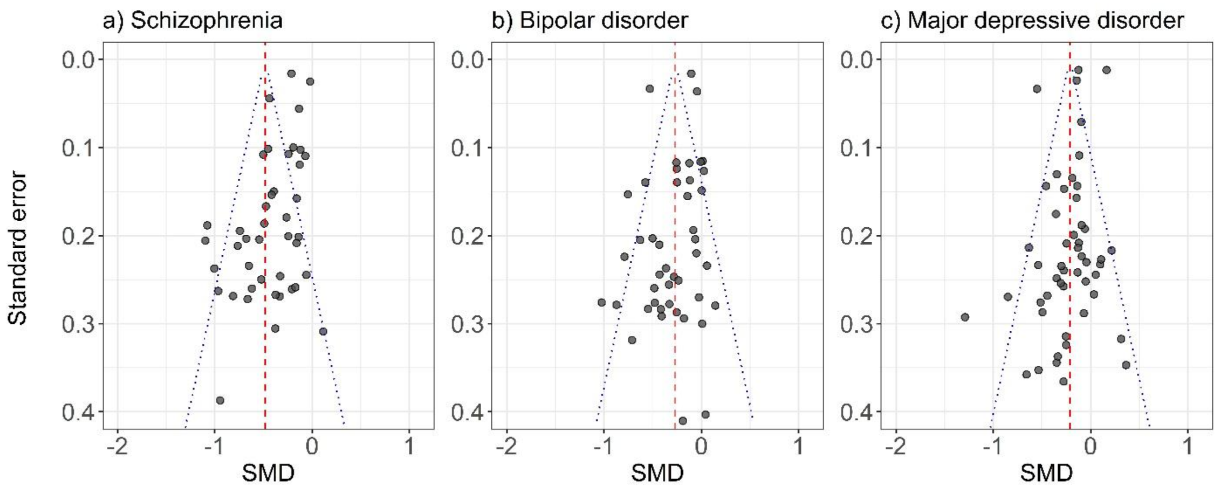

SMD: standardized mean difference

eFigure 2. Funnel plots of studies included in the meta-analysis of parental severe mental illness and offspring cognitive outcomes, based on the mean effect estimate per study

eTable 9. Egger's Test for Funnel Plot Asymmetry Based on Mean Estimates per Study

Parental SMI	Studies	z-value	p-value
Schizophrenia	45	-2.8	0.005
Bipolar disorder	44	-1.5	0.134
Major depressive disorder	51	-1.23	0.217

Note: A p-value < 0.05 suggests significant asymmetry.

eTable 10. Publication bias test using PET regression approach.

Disorder	No of studies	No of estimates	β (SE term)	SE	p-value	I^2
Schizophrenia	45	450	−2.18	0.93	0.032	88.6
Bipolar disorder	44	451	−1.16	0.55	0.050	89.1
Major depressive disorder	51	259	−0.65	0.41	0.136	93.3

SE: standard error

PEP regression model: smd ~ 1 + se, estimated using robust variance estimation (RVE) with small-sample corrections (ρ = 0.80).

References

1. Andreassen AK, Lambek R, Hemager N, et al. Working memory heterogeneity from age 7 to 11 in children at familial high risk of schizophrenia or bipolar disorder- The Danish High Risk and Resilience Study. *Journal of Affective Disorders.* 2023;332:318-326.
2. Hemager N, Christiani CJ, Thorup AAE, et al. Neurocognitive heterogeneity in 7-year-old children at familial high risk of schizophrenia or bipolar disorder: The Danish high risk and resilience study - VIA 7. *Journal of Affective Disorders.* 2022;302:214-223.
3. Auerbach JG, Hans S, Marcus J. Neurobehavioral functioning and social behavior of children at risk for schizophrenia. *Israel Journal of Psychiatry & Related Sciences.* 1993;30(1):40-49.
4. Henin A, Biederman J, Mick E, et al. Psychopathology in the offspring of parents with bipolar disorder: A controlled study. *Biological Psychiatry.* 2005;58(7):554-561.
5. Ayano G, Betts K, Dachew BA, et al. Academic performance in adolescent offspring of mothers with prenatal and perinatal psychiatric hospitalizations: A register-based, data linkage, cohort study. *Psychiatry Res.* 2023;319:114946.

6. Ingstrup KG, Laursen TM, Bergink V, et al. Association of timing of onset of maternal mental disorders with completion of primary education in offspring. *JAMA Psychiatry.* 2019;76(7):761-762.
7. Bakshi N, Pruitt P, Radwan J, et al. Inefficiently increased anterior cingulate modulation of cortical systems during working memory in young offspring of schizophrenia patients. *Journal of Psychiatric Research.* 2011;45(8):1067-1076.
8. Jerlang Christiani CA, Mollegaard Jepsen JR, Thorup A, et al. Social cognition in offspring schizophrenia and bipolar disorder. *European Archives of Psychiatry and Clinical Neuroscience.* 2015;1):S86.
9. Barbour T, Murphy E, Pruitt P, et al. Reduced intra-amygdala activity to positively valenced faces in adolescent schizophrenia offspring. *Schizophrenia Research.* 2010;123(2):126-136.
10. Johnsen LK, Larsen KM, Fuglsang SA, et al. Executive Control and Associated Brain Activity in Children With Familial High-Risk of Schizophrenia or Bipolar Disorder: A Danish Register-based Study. *Schizophrenia Bulletin.* 2023;50(3):567-578.
11. Bauer IE, Suchting R, Van Rheenen TE, et al. The use of component-wise gradient boosting to assess the possible role of cognitive measures as markers of vulnerability to pediatric bipolar disorder. *Cognitive Neuropsychiatry.* 2019;24(2):93-107.
12. Johnson KC, LaPrairie JL, Brennan PA, et al. Prenatal antipsychotic exposure and neuromotor performance during infancy. *Archives of General Psychiatry.* 2012;69(8):787-794.
13. Bell MF, Glauert R, Roos LL, et al. Examining the relationship between maternal mental health-related hospital admissions and childhood developmental vulnerability at school entry in Canada and Australia. *BJPsych Open.* 2023;9:7.
14. Kaplan PS, Danko CM, Diaz A, et al. An associative learning deficit in 1-year-old infants of depressed mothers: role of depression duration. *Infant Behavior & Development.* 2011;34(1):35-44.
15. Biederman J, Uchida M, Chai X, et al. Altered intrinsic functional brain architecture in children at familial risk of major depression. *European Neuropsychopharmacology.* 2016;26(Supplement 2):S714-S715.
16. Kauffman C, Grunebaum H, Cohler B, et al. Superkids: competent children of psychotic mothers. *American Journal of Psychiatry.* 1979;136(11):1398-1402.
17. Bilu Y, Amit G, Gueron-Sela N, et al. Maternal psychiatric admissions and Child developmental delays: A nationwide cohort study. *Archives of Women's Mental Health.* 2025.
18. Kavanaugh BC, Legere C, Vigne M, et al. The Tower of London task in children and adolescents with neuropsychiatric disorders. *Child Neuropsychology.* 2025;31(2):239-254.
19. Blain SD, Kent JS, Peyromaure de Bord CA, et al. Social cognitive deficits in schizophrenia, bipolar disorder, and first-degree relatives: A large-sample, multi-task investigation. *Psychiatry Research.* 2025;352:116694.
20. Kenar J, Citak S, Ceylan ME, et al. Comparison of neurocognitive functions of offspring of schizophrenic, bipolar and controls. *Bulletin of Clinical Psychopharmacology.* 2013;23(Supplement 1):S187.
21. Bohon C, Garber J, Horowitz JL. Predicting school dropout and adolescent sexual behavior in offspring of depressed and nondepressed mothers. *J Am Acad Child Adolesc Psychiatry.* 2007;46(1):15-24.
22. Kestenbaum CJ. Children at risk for schizophrenia. *American Journal of Psychotherapy.* 1980;34(2):164-177.
23. Boisvert M, Dugre JR, Potvin S. Brief Report: Intact Cognitive Functions in Children and Adolescents of Parents With Mood Disorders. *Early Intervention in Psychiatry.* 2025;19(4):e70042.
24. Knudsen CB, Hemager N, Greve AN, et al. Neurocognitive Development in Children at Familial High Risk of Schizophrenia or Bipolar Disorder. *JAMA Psychiatry.* 2022;79(6):589-599.
25. Bora E, Can G, Ildiz A, et al. Neurocognitive heterogeneity in young offspring of patients with bipolar disorder: The effect of putative clinical stages. *Journal of Affective Disorders.* 2019;257:130-135.
26. Knudsen CB, Hemager N, Jepsen JRM, et al. Early Childhood Neurocognition in Relation to Middle Childhood Psychotic Experiences in Children at Familial High Risk of Schizophrenia or Bipolar Disorder and Population-Based Controls: The Danish High Risk and Resilience Study. *Schizophrenia Bulletin.* 2022;49(3):756-767.
27. Bornstein MH, Henry LM, Manian N. Language development in children of clinically depressed mothers in remission: Early experience effects. *Developmental Psychology.* 2021;57(6):876-887.
28. Knudsen CB, Greve AN, Jepsen JRM, et al. Neurocognitive Subgroups in Children at Familial High-risk of Schizophrenia or Bipolar disorder: Subgroup Membership Stability or Change From Age 7 to 11-The Danish High Risk and Resilience Study. *Schizophrenia Bulletin.* 2023;49(1):185-195.
29. Boukhari G, Mnif L, Chakroun M, et al. Are neurocognitive functions altered in descendants of parents with bipolar disorder? *European Psychiatry.* 2019;56(Supplement 1):S26-S27.
30. Lai TJ, Liu X, Guo YL, et al. Neurocognitive impairments in nonpsychotic parents of children with schizophrenia and attention-deficit/hyperactivity disorder: The University of California, Los Angeles family study. *Archives of General Psychiatry.* 2002;59(11):1053-1060.

31. Boukhari G, Mnif L, Chakroun M, et al. Neurocognitive functions in descendants at familial high risk for schizophrenia: what's new? *European Psychiatry.* 2019;56(Supplement 1):S499.
32. Landau R, Harth P, Othnay N, et al. The influence of psychotic parents on their children's development. *American Journal of Psychiatry.* 1972;129(1):38-43.
33. Brennan PA, Hammen C, Andersen MJ, et al. Chronicity, severity, and timing of maternal depressive symptoms: relationships with child outcomes at age 5. *Developmental Psychology.* 2000;36(6):759-766.
34. Le HT, Nguyen HT. Parental health and children's cognitive and noncognitive development: New evidence from the longitudinal survey of Australian children. *Health Economics.* 2017;26(12):1767-1788.
35. Brunovsky M, Horacek J, Viktorinova M, et al. 7.9 Offspring of Parents with Bipolar Disorder Are Characterized by Psychopathological and Neurophysiological but Not Neurocognitive Aberrations. *Journal of the American Academy of Child and Adolescent Psychiatry.* 2020;59(10 Supplement):S170-S171.
36. Lin K, Xu G, Wong NML, et al. A Multi-Dimensional and Integrative Approach to Examining the High-Risk and Ultra-High-Risk Stages of Bipolar Disorder. *eBioMedicine.* 2015;2(8):919-928.
37. Burger M, Einspieler C, Niehaus DJH, et al. Maternal mental health and infant neurodevelopment at 6 months in a low-income South African cohort. *Infant Mental Health Journal.* 2022;43(6):849-863.
38. Liu X, Trabjerg BB, Munk-Olsen T, et al. Association of Maternal Antipsychotic Prescription during Pregnancy with Standardized Test Scores of Schoolchildren in Denmark. *JAMA Internal Medicine.* 2022;182(10):1035-1043.
39. Burger M, Einspieler C, Jordaan ER, et al. Persistent Maternal Mental Health Disorders and Toddler Neurodevelopment at 18 Months: Longitudinal Follow-up of a Low-Income South African Cohort. *International Journal of Environmental Research and Public Health.* 2023;20(12) (no pagination).
40. Liu Z, Lu W, Zou W, et al. A Preliminary Study of Brain Developmental Features of Bipolar Disorder Familial Risk and Subthreshold Symptoms. *Biological Psychiatry: Cognitive Neuroscience and Neuroimaging.* 2025;10(7):769-780.
41. Camprodon-Boadas P, Rosa-Justicia M, Sugranyes G, et al. P.0758 Cognitive reserve and its correlates in child and adolescent offspring of patients diagnosed with schizophrenia or bipolar disorder. *European Neuropsychopharmacology.* 2021;53(Supplement 1):S552.
42. Marcus J, Hans SL, Auerbach JG, et al. Children at risk for schizophrenia: the Jerusalem Infant Development Study. II. Neurobehavioral deficits at school age. *Archives of General Psychiatry.* 1993;50(10):797-809.
43. Can GS, Ulas G, Ildiz A, et al. Neurocognition and neuroimaging features in offspring of Parents with bipolar disorder: A comparative high-risk study. *Psychiatry and Clinical Psychopharmacology.* 2018;28(Supplement 1):32-33.
44. McCormack C, Green MJ, Rowland JE, et al. Neuropsychological and social cognitive function in young people at genetic risk of bipolar disorder. *Psychological Medicine.* 2016;46(4):745-758.
45. Caplan HL, Cogill SR, Alexandra H, et al. Maternal depression and the emotional development of the child. *British Journal of Psychiatry.* 1989;154:818-822.
46. McDonough-Ryan P. *The neuropsychological characterization of children of parents with bipolar disorder* 2001.
47. Christiani CJ, Mollegaard Jepsen JR, Thorup AA, et al. Social cognition, language and social functioning in children with genetic high risk for developing schizophrenia and bipolar disorder. *European Archives of Psychiatry and Clinical Neuroscience.* 2013;1):S102.
48. McNeil TF, Harty B, Blennow G, et al. Neuromotor deviation in offspring of psychotic mothers: a selective developmental deficiency in two groups of children at heightened psychiatric risk? *Journal of Psychiatric Research.* 1993;27(1):39-54.
49. Christiani C, Jepsen JR, Thorup A, et al. Social cognition, language, and social functioning in n offspring with familial high risk for developing schizophrenia and bipolar disorder. *Early Intervention in Psychiatry.* 2014;1):111.
50. Meyer A, Bress JN, Hajcak G, et al. Maternal Depression Is Related to Reduced Error-Related Brain Activity in Child and Adolescent Offspring. *Journal of Clinical Child & Adolescent Psychology.* 2018;47(2):324-335.
51. Christiani CAJ, Jepsen JR, Thorup AAE, et al. Social cognition, language, and social functioning in 7 year old children with familial high risk for developing schizophrenia spectrum disorder or bipolar disorder. Part of the high risk and resilience study - Via 7. *Schizophrenia Research.* 2014;1):S142-S143.
52. Misiak B, Szmida E, Karpiński P, et al. Lower LINE-1 Methylation in First-Episode Schizophrenia Patients with the History of Childhood Trauma. *Epigenomics.* 2015;7(8):1275-1285.
53. Christiani CJ, Jepsen JRM, Thorup A, et al. Social Cognition, Language, and Social Behavior in 7-Year-Old Children at Familial High-Risk of Developing Schizophrenia or Bipolar Disorder: The Danish High Risk and Resilience Study VIA 7-A Population-Based Cohort Study. *Schizophrenia Bulletin.* 2019;45(6):1218-1230.

54. Mubasyiroh R, Suryaputri IY, Bintari DR, et al. Association of parental depression with educational outcomes in Indonesian children aged 6-12 years: A cross-sectional study. *Malaysian Family Physician.* 2025;20:68.
55. Christiani CJ, Hemager N, Ellersgaard D, et al. Heterogeneity of social cognitive and language functions in children at familial high-risk of severe mental illness; The Danish High Risk and Resilience Study VIA 7. *European Child & Adolescent Psychiatry.* 2022;31(6):991-1002.
56. Murage D, Nazarova A, Drobinin V, et al. Familial risk of major mood disorders and brain functional connectivity in the default mode, cognitive executive, and salience networks. *Journal of Psychiatry and Neuroscience.* 2025;50(3):E181-E193.
57. Curl C, Hazelgrove K, Biaggi A, et al. Antenatal psychopathology and infant outcomes: The role of maternal antenatal immune system. *Psychoneuroendocrinology.* 2025;172(Supplement):107314.
58. Murphy V, Short S, Cornea E, et al. Early brain and cognitive development in children at risk for schizophrenia. *Schizophrenia Bulletin.* 2018;44(Supplement 1):S103-S104.
59. D'Angelo EJ. Conceptual disorganization in children at risk for schizophrenia. *Psychopathology.* 1993;26(3-4):195-202.
60. Nayberg E, Macbeth A, Gumley A, et al. Early cognitive and motor development in offspring of mothers with psychotic disorders. *European Archives of Psychiatry and Clinical Neuroscience.* 2017;267(1 Supplement 1):S42.
61. Dave S, Sherr L, Senior R, et al. Major paternal depression and child consultation for developmental and behavioural problems. *British Journal of General Practice.* 2009;59(560):180-185.
62. Ng R. *Cognitive and emotional sequelae of exposure to maternal depression: Memory functioning as a neuropsychological correlate of internalizing symptomatology*, University of Minnesota; 2018.
63. de la Serna E, Baeza I, Andrés S, et al. Comparison between young siblings and offspring of subjects with schizophrenia: Clinical and neuropsychological characteristics. *Schizophrenia Research.* 2011;131(1):35-42.
64. Ng L, Fyfe A, Wang Z, et al. The one-hundred-women study: characteristics of New Zealand women with severe mental illness. *New Zealand Medical Journal.* 2025;138(1621):13-33.
65. de la Serna E, Vila M, Sanchez-Gistau V, et al. Neuropsychological characteristics of child and adolescent offspring of patients with bipolar disorder. *Progress in Neuro-Psychopharmacology & Biological Psychiatry.* 2016;65:54-59.
66. Noniyeva Y. *The relationships between neuropsychological and behavioral, personal, and social functioning of healthy offspring with family history of bipolar disorder*, Palo Alto University; 2018.
67. de la Serna E, Sugranyes G, Sanchez-Gistau V, et al. Neuropsychological characteristics of child and adolescent offspring of patients with schizophrenia or bipolar disorder. *Schizophrenia Research.* 2017;183:110-115.
68. Novak T, Sebela A, Mohaplova M, et al. Neuropsychological functioning in child and adolescent ofspring of parents with bipolar disorder. *Annals of General Psychiatry Conference: 2nd Congress on Evidence Based Mental Health: From Research to Clinical Practice Kavala Greece.* 2018;17(Supplement 1).
69. de la Serna E, Baeza I, Toro J, et al. Relationship between clinical and neuropsychological characteristics in child and adolescent first degree relatives of subjects with schizophrenia. *Schizophrenia Research.* 2010;116(2-3):159-167.
70. Nulman I, Koren G, Rovet J, et al. Neurodevelopment of children following prenatal exposure to venlafaxine, selective serotonin reuptake inhibitors, or untreated maternal depression. *American Journal of Psychiatry.* 2012;169(11):1165-1174.
71. De La Serna E, Noguera A, Baeza I, et al. Clinical and neuropsychological characteristics in child and adolescent at high risk for schizophrenia: Comparative study between first degree relatives. *Early Intervention in Psychiatry.* 2010;1):97.
72. Pagliaccio D, Alqueza KL, Marsh R, et al. Brain Volume Abnormalities in Youth at High Risk for Depression: Adolescent Brain and Cognitive Development Study. *Journal of the American Academy of Child & Adolescent Psychiatry.* 2020;59(10):1178-1188.
73. De La Serna E. Neuropsychological characteristics of children and adolescent offspring of patients diagnosed with schizophrenia or bipolar disorder: A two-year followup study. *Journal of the American Academy of Child and Adolescent Psychiatry.* 2017;56(10):S314.
74. Pariante C. Impact of Maternal Mental Illness on the Mothers and the Children: A 7-9 Years Follow-Up Study. *Psychoneuroendocrinology.* 2023;Conference: ISPNE 2022 . Chicago United States. 153(Supplement) (no pagination).
75. De la Serna E, Sugranyes G, Moreno D, et al. Neuropsychological characteristics of children and adolescent offspring of patients diagnosed with schizophrenia or bipolar disorder. *European Neuropsychopharmacology.* 2019;29(Supplement 1):S102-S103.

76. Parrilla-Escobar MA, Quintana-Velasco JL, Maniega-Rubio A, et al. Cognitive and motor alterations in children attending a psychiatric clinic in relation to schizophrenia spectrum family antecedents and thought problems. *European Journal of Psychiatry.* 2024;38(3) (no pagination).
77. De Zwarte S, Brouwer R, Hillegers M, et al. Brain abnormalities and iq in offspring, siblings, co-twins, and parents of patients with schizophrenia. *Schizophrenia Bulletin.* 2017;43(Supplement 1):S48-S49.
78. Patil S. Assessment and Comparison of Cognitive Function in Offspring of Patients Diagnosed with Schizophrenia and Alcohol Dependence Syndrome. *International Journal of Medical and Pharmaceutical Research.* 2025;6(5):1656-1669.
79. De Zwarte S, Brouwer R, Tsouli A, et al. Brain abnormalities and cognitive deficits in first-degree relatives of patients with schizophrenia. *Biological Psychiatry.* 2018;83(9 Supplement 1):S325.
80. Peredo R, Gagne AM, Gilbert E, et al. Electroretinography may reveal cognitive impairment among a cohort of subjects at risk of a major psychiatric disorder. *Psychiatry Research.* 2020;291(no pagination).
81. de Zwarte SMC, Brouwer RM, Tsouli A, et al. Running in the Family? Structural Brain Abnormalities and IQ in Offspring, Siblings, Parents, and Co-twins of Patients with Schizophrenia. *Schizophrenia Bulletin.* 2018;45(6):1209-1217.
82. Prasad KM, Sanders R, Sweeney J, et al. Neurological abnormalities among offspring of persons with schizophrenia: relation to premorbid psychopathology. *Schizophrenia Research.* 2009;108(1-3):163-169.
83. de Zwarte SMC, Brouwer RM, Agartz I, et al. Intelligence, educational attainment, and brain structure in those at familial high-risk for schizophrenia or bipolar disorder. *Human Brain Mapping.* 2022;43(1):414-430.
84. Razaz N, Joseph KS, Boyce WT, et al. Children of chronically ill parents: Relationship between parental multiple sclerosis and childhood developmental health. *Multiple Sclerosis.* 2016;22(11):1452-1462.
85. Dickson H, Cullen AE, Jones R, et al. Trajectories of cognitive development during adolescence among youth at-risk for schizophrenia. *Journal of Child Psychology & Psychiatry & Allied Disciplines.* 2018;59(11):1215-1224.
86. Remberk B, Namyslowska I. Cognitive impairment and formal thought disorders in adolescent schizophrenia spectrum patients and their parents. *European Neuropsychopharmacology.* 2011;3):S465-S466.
87. Diwadkar VA, Pruitt P, Zhang A, et al. The neural correlates of performance in adolescents at risk for schizophrenia: Inefficiently increased cortico-striatal responses measured with fMRI. *Journal of Psychiatric Research.* 2012;46(1):12-21.
88. Rogers AM, Youssef GJ, Teague S, et al. Association of maternal and paternal perinatal depression and anxiety with infant development: A longitudinal study. *Journal of Affective Disorders.* 2023;338:278-288.
89. Diwadkar V. Brain network profiles for attention, memory and emotion in children and adolescents at risk for schizophrenia. *Schizophrenia Bulletin.* 2013;1):S153.
90. Rolf JE. The social and academic competence of children vulnerable to schizophrenia and other behavior pathologies. *Journal of Abnormal Psychology.* 1972;80(3):225-243.
91. Duffy A, Hajek T, Alda M, et al. Neurocognitive functioning in the early stages of bipolar disorder: visual backward masking performance in high risk subjects. *European Archives of Psychiatry & Clinical Neuroscience.* 2009;259(5):263-269.
92. Ross RG, Wagner B, Heinlein S, et al. The stability of inhibitory and working memory deficits in children and adolescents who are children of parents with schizophrenia. *Schizophrenia Bulletin.* 2008;34(1):47-51.
93. Eriksen HL, Kesmodel US, Pedersen LH, et al. No association between prenatal exposure to psychotropics and intelligence at age five. *Acta Obstet Gynecol Scand.* 2015;94(5):501-507.
94. Salisbury AL, Wisner KL, Pearlstein T, et al. Newborn neurobehavioral patterns are differentially related to prenatal maternal major depressive disorder and serotonin reuptake inhibitor treatment. *Depression & Anxiety.* 2011;28(11):1008-1019.
95. Espie J, Jones SH, Vance YH, et al. Brief report: A family risk study exploring bipolar spectrum problems and cognitive biases in adolescent children of bipolar parents. *Journal of Adolescence.* 2012;35(3):769-772.
96. Schrijver L, Robakis TK, Kamperman AM, et al. Neurodevelopment in school-aged children after intrauterine exposure to antipsychotics. *Acta Psychiatrica Scandinavica.* 2023;147(1):43-53.
97. Florsheim J, Peterfreund O. The intelligence of parents of psychotic children. *Journal of Autism & Childhood Schizophrenia.* 1974;4(1):61-70.
98. Schubert EW, McNeil TF. Neurobehavioral deficits in young adult offspring with heightened risk for psychosis who developed schizophrenia-spectrum disorder. *Schizophrenia Research.* 2007;94(1-3):107-113.
99. Fricke J, Bolster M, Icke K, et al. Psychiatric disorders in psychosocially burdened mothers with young children: a population-based cohort study in Germany. *Frontiers in Psychiatry.* 2025;16:1477336.

100. Seidman LJ, Cherkerzian S, Goldstein JM, et al. Neuropsychological performance and family history in children at age 7 who develop adult schizophrenia or bipolar psychosis in the New England Family Studies. *Psychological Medicine.* 2013;43(1):119-131.
101. Friedman EH. Neurocognitive deficits in infants of mothers with schizophrenia. *British Journal of Psychiatry.* 2000;177:564-565.
102. Sharma A, Barron E, Le Couteur J, et al. Neurocognitive function in children of adults with bipolar disorder: A UK study. *Bipolar Disorders.* 2011;1):88-89.
103. Gilbert E, Paccalet T, Jomphe V, et al. Change and stability in cognitive trajectories from childhood to late adolescence in young offspring at genetic risk of schizophrenia and mood disorder: Implications for the risk status. *Schizophrenia Bulletin.* 2018;44(Supplement 1):S142.
104. Short S, Goldman BD, Styner M, et al. Infant brain structure and cognitive development in children at high-risk for schizophrenia. *Schizophrenia Bulletin.* 2013;1):S248.
105. Gilmore J. Cortical thickness, surface area and cognitive development in very young children at risk for schizophrenia. *Neuropsychopharmacology.* 2017;43(Supplement 1):S20-S21.
106. Sidorchuk A, Brander G, Perez-Vigil A, et al. One versus two biological parents with mental disorders: Relationship to educational attainment in the next generation. *Psychological Medicine.* 2023;53(15):7025-7041.
107. Goetz M, Sebela A, Mohaplova M, et al. Psychiatric Disorders and Quality of Life in the Offspring of Parents with Bipolar Disorder. *J Child Adolesc Psychopharmacol.* 2017;27(6):483-493.
108. Siegel-Ramsay J, Layfield S, Bichlmeier A, et al. Neurodevelopmental Patterns During Peer Exclusion in Youth at Increased Familial Risk for Bipolar I Disorder. *Bipolar Disorders.* 2025;27(Supplement 1):S52.
109. Goetz M, Novak T, Viktorinova M, et al. Not Neuropsychological Functioning But Temperament Differentiates Children and Adolescents at Risk of Bipolar Disorder From Controls. *Journal of the American Academy of Child and Adolescent Psychiatry.* 2018;57(10 Supplement):S167.
110. Sinha SK, Kishore MT, Thippeswamy H, et al. Adverse effects and short-term developmental outcomes of infants exposed to atypical antipsychotics during breastfeeding. *Indian Journal of Psychiatry.* 2021;63(1):52-57.
111. Gregersen M, Rohd SB, Jepsen JRM, et al. Jumping to Conclusions and Its Associations With Psychotic Experiences in Preadolescent Children at Familial High Risk of Schizophrenia or Bipolar Disorder-The Danish High Risk and Resilience Study, VIA 11. *Schizophrenia Bulletin.* 2022;48(6):1363-1372.
112. Skurtveit S, Selmer R, Roth C, et al. Prenatal exposure to antidepressants and language competence at age three: results from a large population-based pregnancy cohort in Norway. *BJOG: An International Journal of Obstetrics & Gynaecology.* 2014;121(13):1621-1631.
113. Greve AN, Hemager N, Mortensen EL, et al. Comparing cognition in parents with schizophrenia or bipolar disorder and their 7-year-old offspring. *Psychiatry Research.* 2024;340:116112.
114. Stephens R, Cornea E, Short S, et al. Early Cognitive and Clinical Outcomes in Children at High Familial Risk for Schizophrenia. *Biological Psychiatry.* 2021;89(9 Supplement):S216-S217.
115. Greve AN, Jepsen JRM, Mortensen EL, et al. Transmission of intelligence, working memory, and processing speed from parents to their seven-year-old offspring is function specific in families with schizophrenia or bipolar disorder. *Schizophrenia Research.* 2022;246:195-201.
116. Stephens RL, Leavitt I, Cornea E, et al. Early cognitive development and psychopathology in children at familial high risk for schizophrenia. *Schizophr Res.* 2024;271:262-270.
117. Grunebaum H, Cohler BJ, Kauffman C, et al. Children of depressed and schizophrenic mothers. *Child Psychiatry & Human Development.* 1978;8(4):219-228.
118. Sugranyes G, De La Serna E, Borras R, et al. Clinical, cognitive and imaging evidence of a neurodevelopmental continuum in of fspring of probands with schizophrenia and bipolar disorder. *European Neuropsychopharmacology.* 2017;27(Supplement 4):S912.
119. Gumley A, Davidsen K, MacBeth A, et al. Evidence of early developmental risk in offspring of parents with psychotic disorders. *European Archives of Psychiatry and Clinical Neuroscience.* 2017;267(1 Supplement 1):S42.
120. Sugranyes G, de la Serna E, Borras R, et al. Clinical, Cognitive, and Neuroimaging Evidence of a Neurodevelopmental Continuum in Offspring of Probands With Schizophrenia and Bipolar Disorder. *Schizophrenia Bulletin.* 2017;43(6):1208-1219.
121. Gunnarsdottir ED, Hallgren J, Hultman CM, et al. Risk of neurological, eye and ear disease in offspring to parents with schizophrenia or depression compared with offspring to healthy parents. *Psychological Medicine.* 2018;48(16):2710-2716.
122. Sugranyes G, de la Serna E, Ilzarbe D, et al. Brain structural trajectories in youth at familial risk for schizophrenia or bipolar disorder according to development of psychosis spectrum symptoms. *Journal of Child Psychology and Psychiatry.* 2021;62(6):780-789.

123. Gustafsson HC, Goodman SH, Feng T, et al. Major depressive disorder during pregnancy: Psychiatric medications have minimal effects on the fetus and infant yet development is compromised. *Development & Psychopathology.* 2018;30(3):773-785.
124. Thorup AAE, Jepsen JR, Plessen K, et al. Copenhagen high risk study II: Childhood and development. 7-year old children of parents with schizophrenia. *Schizophrenia Research.* 2012;1):S302-S303.
125. Hankin BL, Schweizer TH, Young JF. Trajectories of rumination and negative cognitive style from late childhood through adolescence: Modeling normative growth patterns and predicting cognitive vulnerabilities. *Clinical Psychological Science.* 2025;13(6):1126-1145.
126. Thorup AA, Hemager N, Ellersgaard DV, et al. The danish high-risk and resilience study - Via 7 - ative cohorte study of 522 7 years old children born to parents diagnosed with schizophrenia or bipolar disorder - Results on psychopathology, cognition and living conditions. *Schizophrenia Bulletin.* 2018;44(Supplement 1):S300.
127. Hans SL, Marcus J, Nuechterlein KH, et al. Neurobehavioral deficits at adolescence in children at risk for schizophrenia: The Jerusalem Infant Development Study. *Archives of General Psychiatry.* 1999;56(8):741-748.
128. Tognin S, Catalan A, Aymerich C, et al. Association between Adverse Childhood Experiences and long-term outcomes in people at Clinical High-Risk for Psychosis. *Schizophrenia.* 2025;11(1):23.
129. Hans SL, Auerbach JG, Auerbach AG, et al. Development from Birth to Adolescence of Children At-Rlsk for Schizophrenia. *Journal of Child and Adolescent Psychopharmacology.* 2005;15(3):384-394.
130. Tsypes A, Gibb BE. Cognitive vulnerabilities and development of suicidal thinking in children of depressed mothers: A longitudinal investigation. *Psychiatry Research.* 2016;239:99-104.
131. Hans SL, Auerbach JG, Nuechterlein KH, et al. Neurodevelopmental factors associated with schizotypal symptoms among adolescents at risk for schizophrenia. *Development and Psychopathology.* 2009;21(4):1195-1210.
132. Ucok Demir N, Perdahli Fis N. Evaluation of social cognition in children of bipolar parents. *European Child and Adolescent Psychiatry.* 2015;1):S253.
133. Harjan A. Developmental delay in offspring of parents with affective disorders and depression: psycho-social sequels or a constitutional state? *Acta Paedopsychiatrica.* 1989;52(4):287-297.
134. Valli I, De La Serna E, Borràs R, et al. Cognitive heterogeneity in the offspring of patients with schizophrenia or bipolar disorder: A cluster analysis across family risk. *Journal of Affective Disorders.* 2021;282:757-765.
135. Hemager N, Jepsen JR, Thorup A, et al. Neurocognition in 7-year-old children of parents with schizophrenia or bipolar affective disorder. Part of the Danish High Risk and Resilience Study-VIA 7. *Early Intervention in Psychiatry.* 2012;6(SUPPL.1):43.
136. Van Haren N. Brain abnormalities and IQ in offspring related to those in siblings, co-twins, and parents of patients with schizophrenia. *Journal of the American Academy of Child and Adolescent Psychiatry.* 2017;56(10):S313.
137. Hemager N, Mollegaard Jepsen JR, Thorup A, et al. Neurocognitive profiles in 7-year-old offspring of parents with schizophrenia or with bipolar disorder-part of the Danish High Risk and Resilience Study "VIA 7". *European Archives of Psychiatry and Clinical Neuroscience.* 2013;1):S103.
138. Ver Loren van Themaat AH, Uddin MJ, Christiani CJ, et al. Odor identification in 7-year-old children at familial high risk of schizophrenia or bipolar disorder - the Danish high risk and resilience study VIA 7. *Schizophrenia Research.* 2020;216:77-84.
139. Hemager N, Jepsen JRM, Thorup AAE, et al. Neurocognitive profiles in 7-year-old offspring of parents with schizophrenia or bipolar disorder. Part of the high risk and resilience study - Via 7. *Schizophrenia Research.* 2014;1):S143-S144.
140. Wisner KL. Impact of prenatal exposure to SSRIs or maternal depression disorder on infant developmental outcomes. *European Neuropsychopharmacology.* 2015;2):S156.
141. Hemager N, Plessen K, Thorup AA, et al. Neurocognition in 7-year-old children of parents with schizophrenia or bipolar disorder. *Schizophrenia Bulletin.* 2018;44(Supplement 1):S245-S246.
142. Woody ML, Tsypes A, Burkhouse KL, et al. Development of Overgeneral Autobiographical Memory in Offspring of Depressed Mothers. *Journal of Clinical Child & Adolescent Psychology.* 2022;51(1):73-84.
143. Hemager N, Vangkilde S, Thorup A, et al. Visual attention in 7-year-old children at familial high risk of schizophrenia or bipolar disorder: The Danish high risk and resilience study VIA 7. *Journal of Affective Disorders.* 2019;258:56-65.
144. Xie Y, Zou W, Shang Y, et al. Cognitive and neural abnormalities: working memory deficits in bipolar disorder offspring. *Psychological medicine.* 2025;55:e130.
145. Hemager N, Jepsen JRM, Thorup A, et al. Decision making and its associations to neurocognitive functions, psychopathology, and the home environment in seven-year-old children at familial high risk of

schizophrenia or bipolar disorder: The Danish High Risk and Resilience Study VIA 7. *Journal of Affective Disorders.* 2021;281:609-617.
146. Zahn-Waxler C, Chapman M, Cummings EM. Cognitive and social development in infants and toddlers with a bipolar parent. *Child Psychiatry & Human Development.* 1984;15(2):75-85.
147. Anderson CA, Hammen CL. Psychosocial outcomes of children of unipolar depressed, bipolar, medically ill, and normal women: a longitudinal study. *Journal of Consulting & Clinical Psychology.* 1993;61(3):448-454.
148. Andreassen AK, Lambek R, Greve A, et al. The development in rating-based executive functions in children at familial high risk of schizophrenia or bipolar disorder from age 7 to age 11: the Danish high risk and resilience study. *European Child & Adolescent Psychiatry.* 2024;33(2):549-560.
149. Arici Sagliyan G, Cetin FH, Akyurek F, et al. Arginine metabolism and neurocognitive impairment in offspring of bipolar parents: a high-risk case-control study. *Frontiers in Psychiatry.* 2025;16:1511397.
150. Asarnow LD, Thompson RJ, Joormann J, et al. Children at risk for depression: memory biases, self-schemas, and genotypic variation. *J Affect Disord.* 2014;159:66-72.
151. Auerbach JG, Hans SL, Zhang Y. Neurocognitive functioning in adult and adolescent offspring of parents with schizophrenia. *Schizophrenia Research.* 2022;248:300-308.
152. Burger M, Einspieler C, Niehaus DJH, et al. Maternal mental health and infant neurodevelopment at 6 months in a low-income South African cohort. *Infant Mental Health Journal.* 2022;43(6):849-863.
153. Burton BK, Vangkilde S, Petersen A, et al. Sustained Attention and Interference Control Among 7-Year-Old Children With a Familial High Risk of Schizophrenia or Bipolar Disorder—A Nationwide Observational Cohort Study. *Biological Psychiatry: Cognitive Neuroscience and Neuroimaging.* 2018;3(8):704-712.
154. Camprodon-Boadas P, Rosa-Justicia M, Sugranyes G, et al. Cognitive reserve and its correlates in child and adolescent offspring of patients diagnosed with schizophrenia or bipolar disorder. *European Child & Adolescent Psychiatry.* 2023;32(8):1463-1473.
155. Can G, Bora E, Ildiz A, et al. Neurocognition in young offspring of individuals with bipolar disorder: The role of co-existing familial and clinical high-risk for bipolar disorder. *Psychiatry Research.* 2019;281:112565.
156. Chai XJ, Hirshfeld-Becker D, Biederman J, et al. Altered Intrinsic Functional Brain Architecture in Children at Familial Risk of Major Depression. *Biological Psychiatry.* 2016;80(11):849-858.
157. Christiani CJ, Jepsen JRM, Thorup A, et al. Social Cognition, Language, and Social Behavior in 7-Year-Old Children at Familial High-Risk of Developing Schizophrenia or Bipolar Disorder: The Danish High Risk and Resilience Study VIA 7—A Population-Based Cohort Study. *Schizophrenia Bulletin.* 2019;45(6):1218-1230.
158. Cicchetti D, Rogosch FA, Toth SL. The efficacy of toddler-parent psychotherapy for fostering cognitive development in offspring of depressed mothers. *J Abnorm Child Psychol.* 2000;28(2):135-148.
159. Conroy S, Pariante CM, Marks MN, et al. Maternal psychopathology and infant development at 18 months: the impact of maternal personality disorder and depression. *J Am Acad Child Adolesc Psychiatry.* 2012;51(1):51-61.
160. Davalos DB, Compagnon N, Heinlein S, et al. Neuropsychological deficits in children associated with increased familial risk for schizophrenia. *Schizophrenia Research.* 2004;67(2-3):123-130.
161. De la Serna E, Camprodon-Boadas P, Ilzarbe D, et al. Neuropsychological development in the child and adolescent offspring of patients diagnosed with schizophrenia or bipolar disorder: A two-year follow-up comparative study. *Progress in Neuro-Psychopharmacology & Biological Psychiatry.* 2020;103:109972.
162. Deveci E, Ozan E, Kirpinar I, et al. Neurocognitive functioning in young high-risk offspring having a parent with bipolar I disorder. *Turkish Journal of Medical Sciences.* 2013;43(1):110-117.
163. Diwadkar VA, Goradia D, Hosanagar A, et al. Working memory and attention deficits in adolescent offspring of schizophrenia or bipolar patients: comparing vulnerability markers. *Prog Neuropsychopharmacol Biol Psychiatry.* 2011;35(5):1349-1354.
164. ElSehrawy TMK, Elela EA, Hassan GAM, et al. A study of emotional intelligence in an Egyptian sample of offspring of patients with schizophrenia. *Middle East Current Psychiatry.* 2022;29(1):48.
165. Fattahi Asl A, Ghanizadeh A, Mollazade J, et al. Differences of biased recall memory for emotional information among children and adolescents of mothers with MDD, children and adolescents with MDD, and normal controls. *Psychiatry Research.* 2015;228(2):223-227.
166. Fis NP, Cetin FC, Erturk M, et al. Executive dysfunction in Turkish children at high risk for schizophrenia. *European Child & Adolescent Psychiatry.* 2008;17(7):424-431.
167. Galbally M, Lewis AJ, Buist A. Developmental outcomes of children exposed to antidepressants in pregnancy. *Aust N Z J Psychiatry.* 2011;45(5):393-399.

168. Goetz M, Novak T, Viktorinova M, et al. Neuropsychological Functioning and Temperament Traits in a Czech Sample of Children and Adolescents at Familial Risk of Bipolar Disorder. *Front Psychiatry.* 2019;10:198.
169. Goldstein JM, Seidman LJ, Buka SL, et al. Impact of Genetic Vulnerability and Hypoxia on Overall Intelligence by Age 7 in Offspring at High Risk for Schizophrenia Compared With Affective Psychoses. *Schizophrenia Bulletin.* 2000;26(2):323-334.
170. Goodman SH. Emory University Project on Children of Disturbed Parents. *Schizophr Bull.* 1987;13(3):411-423.
171. Gotlib IH, Traill SK, Montoya RL, et al. Attention and memory biases in the offspring of parents with bipolar disorder: indications from a pilot study. *Journal of Child Psychology & Psychiatry & Allied Disciplines.* 2005;46(1):84-93.
172. Gumustas F, Kutuk EK, Yulaf Y, et al. Psychiatric disorders, developmental, and academic difficulties among children and adolescents at-risk for schizophrenia: A controlled study. *Psychiatry and Clinical Psychopharmacology.* 2018;28(2):142-148.
173. Hanford LC, Sassi RB, Minuzzi L, et al. Cortical thickness in symptomatic and asymptomatic bipolar offspring. *Psychiatry Research: Neuroimaging.* 2016;251:26-33.
174. Hanley GE, Brain U, Oberlander TF. Infant developmental outcomes following prenatal exposure to antidepressants, and maternal depressed mood and positive affect. *Early Hum Dev.* 2013;89(8):519-524.
175. Haren Nv, Nikita S, Koevoets M, et al. Differences in intracranial volume, IQ and psychopathology in young offspring of patients affected with schizophrenia or bipolar disorder. *Schizophrenia Bulletin.* 2018;44(Supplement 1):S225.
176. Hay DF, Pawlby S, Sharp D, et al. Intellectual problems shown by 11-year-old children whose mothers had postnatal depression. *J Child Psychol Psychiatry.* 2001;42(7):871-889.
177. Hemager N, Plessen KJ, Thorup A, et al. Assessment of neurocognitive functions in 7-year-old children at familial high risk for schizophrenia or bipolar disorder the danish high risk and resilience study VIA 7. *JAMA Psychiatry.* 2018;75(8):844-852.
178. Henriksson KM, McNeil TF. Health and development in the first 4 years of life in offspring of women with schizophrenia and affective psychoses: Well-Baby Clinic information. *Schizophrenia Research.* 2004;70(1):39-48.
179. Hirose T, Barnard K. Interactions Between Depressed Mothers and Their Infants: Maternal Verbal Joint Attention and its Effect on the Infant's Cognitive Development. *Early Child Development and Care.* 1997;138(1):83-95.
180. Horton LE, Bridgwater MA, Haas GL. Emotion recognition and social skills in child and adolescent offspring of parents with schizophrenia. *Cognitive Neuropsychiatry.* 2017;22(3):175-185.
181. Howes Vallis E, MacKenzie LE, Rempel S, et al. Visual memory in offspring of parents with mental illness. *Psychiatry Research.* 2020;286(no pagination).
182. Huang H, Fan X, Williamson DE, et al. White matter changes in healthy adolescents at familial risk for unipolar depression: a diffusion tensor imaging study. *Neuropsychopharmacology.* 2011;36(3):684-691.
183. Jundong J, Kuja-Halkola R, Hultman C, et al. Poor school performance in offspring of patients with schizophrenia: what are the mechanisms? *Psychological medicine.* 2012;42(1):111-123.
184. Kersten-Alvarez LE, Hosman CM, Riksen-Walraven JM, et al. Early school outcomes for children of postpartum depressed mothers: comparison with a community sample. *Child Psychiatry Hum Dev.* 2012;43(2):201-218.
185. Kim E, Garrett A, Boucher S, et al. Inhibited Temperament and Hippocampal Volume in Offspring of Parents with Bipolar Disorder. *J Child Adolesc Psychopharmacol.* 2017;27(3):258-265.
186. Klimes-Dougan B, Ronsaville D, Wiggs EA, et al. Neuropsychological functioning in adolescent children of mothers with a history of bipolar or major depressive disorders. *Biological Psychiatry.* 2006;60(9):957-965.
187. Kluczniok D, Hindi Attar C, Fydrich T, et al. Transgenerational effects of maternal depression on affect recognition in children. *Journal of Affective Disorders.* 2016;189:233-239.
188. Kujawa A, Dougherty L, Durbin CE, et al. Emotion recognition in preschool children: associations with maternal depression and early parenting. *Dev Psychopathol.* 2014;26(1):159-170.
189. Levitan RD, Atkinson L, Knight JA, et al. Maternal major depression during early pregnancy is associated with impaired child executive functioning at 4.5 years of age. *American Journal of Obstetrics and Gynecology.* 2024;231(2):246.e241-246.e210.
190. Lin YH, Tsai SJ, Bai YM, et al. Risk of Neurodevelopmental Disorders in Offspring of Parents with Major Depressive Disorder: A Birth Cohort Study. *Journal of Autism and Developmental Disorders.* 2024.
191. Lin YH, Tsai SJ, Bai YM, et al. Risk of Neurodevelopmental Disorders in Offspring of Parents with Major Depressive Disorder: A Birth Cohort Study. *Journal of Autism and Developmental Disorders.* 2025;55(11):4009-4017.

192. Lin A, Di Prinzio P, Young D, et al. Academic performance in children of mothers with schizophrenia and other severe mental illness, and risk for subsequent development of psychosis: A population-based study. *Schizophrenia Bulletin.* 2017;43(1):205-213.
193. Lin K, Lu R, Chen K, et al. Differences in cognitive deficits in individuals with subthreshold syndromes with and without family history of bipolar disorder. *J Psychiatr Res.* 2017;91:177-183.
194. Lopez-Duran NL, Kuhlman KR, George C, et al. Facial emotion expression recognition by children at familial risk for depression: high-risk boys are oversensitive to sadness. *J Child Psychol Psychiatry.* 2013;54(5):565-574.
195. MacKenzie LE, Howes Vallis E, Rempel S, et al. Cognition in offspring of parents with psychotic and non-psychotic severe mental illness. *Journal of Psychiatric Research.* 2020;130:306-312.
196. Maróthi R, Kéri S. Intuitive physics and intuitive psychology ("theory of mind") in offspring of mothers with psychoses. *PeerJ.* 2014;2:e330.
197. Maselko J, Sikander S, Bhalotra S, et al. Effect of an early perinatal depression intervention on long-term child development outcomes: follow-up of the Thinking Healthy Programme randomised controlled trial. *Lancet Psychiatry.* 2015;2(7):609-617.
198. Maziade M, Rouleau N, Gingras N, et al. Shared neurocognitive dysfunctions in young offspring at extreme risk for schizophrenia or bipolar disorder in eastern Quebec multigenerational families. *Schizophrenia Bulletin.* 2009;35(5):919-930.
199. McDonough-Ryan P, DelBello M, Shear PK, et al. Academic and cognitive abilities in children of parents with bipolar disorder: A test of the nonverbal learning disability model. *Journal of Clinical and Experimental Neuropsychology.* 2002;24(3):280-285.
200. Meiser S, Zietlow AL, Reck C, et al. The impact of postpartum depression and anxiety disorders on children's processing of facial emotional expressions at pre-school age. *Arch Womens Ment Health.* 2015;18(5):707-716.
201. Micco JA, Henin A, Biederman J, et al. Executive functioning in offspring at risk for depression and anxiety. *Depression & Anxiety.* 2009;26(9):780-790.
202. Milgrom J, Westley DT, Gemmill AW. The mediating role of maternal responsiveness in some longer term effects of postnatal depression on infant development. *Infant Behavior and Development.* 2004;27(4):443-454.
203. Monk CS, Klein RG, Telzer EH, et al. Amygdala and nucleus accumbens activation to emotional facial expressions in children and adolescents at risk for major depression. *Am J Psychiatry.* 2008;165(1):90-98.
204. Morgan VA, Croft ML, Valuri GM, et al. Intellectual disability and other neuropsychiatric outcomes in high-risk children of mothers with schizophrenia, bipolar disorder and unipolar major depression. *British Journal of Psychiatry.* 2012;200(4):282-289.
205. Murray L, Hipwell A, Hooper R, et al. The cognitive development of 5-year-old children of postnatally depressed mothers. *J Child Psychol Psychiatry.* 1996;37(8):927-935.
206. Murray L, Arteche A, Fearon P, et al. The effects of maternal postnatal depression and child sex on academic performance at age 16 years: a developmental approach. *J Child Psychol Psychiatry.* 2010;51(10):1150-1159.
207. Nulman I, Rovet J, Stewart DE, et al. Child development following exposure to tricyclic antidepressants or fluoxetine throughout fetal life: a prospective, controlled study. *Am J Psychiatry.* 2002;159(11):1889-1895.
208. Oberlander TF, Reebye P, Misri S, et al. Externalizing and attentional behaviors in children of depressed mothers treated with a selective serotonin reuptake inhibitor antidepressant during pregnancy. *Arch Pediatr Adolesc Med.* 2007;161(1):22-29.
209. O'Leary N, Jairaj C, Molloy EJ, et al. Antenatal depression and the impact on infant cognitive, language and motor development at six and twelve months postpartum. *Early Human Development.* 2019;134:41-46.
210. Öner Ö, Munir K. Attentional and neurocognitive characteristics of high-risk offspring of parents with schizophrenia compared with DSM-IV attention deficit hyperactivity disorder children. *Schizophrenia Research.* 2005;76(2):293-299.
211. Osborne S, Biaggi A, Hazelgrove K, et al. Increased maternal inflammation and poorer infant neurobehavioural competencies in women with a history of major depressive disorder from the Psychiatry Research And Motherhood—Depression (PRAM-D) study. *Brain, Behavior, and Immunity.* 2022;99:223-230.
212. Ozan E, Deveci E, Oral M, et al. Neurocognitive functioning in a group of offspring genetically at high-risk for schizophrenia in Eastern Turkey. *Brain Research Bulletin.* 2010;82(3-4):218-223.
213. Patino LR, Adler CM, Mills NP, et al. Conflict monitoring and adaptation in individuals at familial risk for developing bipolar disorder. *Bipolar Disorders.* 2013;15(3):264-271.
214. Pérez-Edgar K, Fox NA, Cohn JF, et al. Behavioral and electrophysiological markers of selective attention in children of parents with a history of depression. *Biol Psychiatry.* 2006;60(10):1131-1138.

215. Pine DS, Klein RG, Mannuzza S, et al. Face-emotion processing in offspring at risk for panic disorder. *J Am Acad Child Adolesc Psychiatry.* 2005;44(7):664-672.
216. Propper L, Sandstrom A, Rempel S, et al. Attention-deficit/hyperactivity disorder and other neurodevelopmental disorders in offspring of parents with depression and bipolar disorder. *Psychological Medicine.* 2023;53(2):559-566.
217. Quevedo LA, Silva RA, Godoy R, et al. The impact of maternal post-partum depression on the language development of children at 12 months. *Child Care Health Dev.* 2012;38(3):420-424.
218. Raju R, Srikar M, Swaminathan D, et al. Maternal Linguistic Input to Infants Born to Mothers With and Without Postpartum Psychosis and Infant Language Skills: A Preliminary Study. *American journal of speech-language pathology.* 2025;34(6):3477-3488.
219. Ranning A, Laursen T, Agerbo E, et al. School performance from primary education in the adolescent offspring of parents with schizophrenia and bipolar disorder- a national, register-based study. *Psychological medicine.* 2018;48(12):1993-2000.
220. Restrepo-Mejia SF, Valencia-Echeverry J, Zapata-Ospina JP, et al. Comparison of the neurocognitive profile of the children of parents with bipolar disorder and controls: A transnational cross-sectional study. *Revista Colombiana De Psiquiatria.* 2023;52(4):320-327.
221. Rieder RO, Broman SH, Rosenthal D. The offspring of schizophrenics. II. Perinatal factors and IQ. *Archives of General Psychiatry.* 1977;34(7):789-799.
222. Rybakowski JK, Permoda-Osip A, Borkowska A. Response to prophylactic lithium in bipolar disorder may be associated with a preservation of executive cognitive functions. *European Neuropsychopharmacology.* 2009;19(11):791-795.
223. Sánchez-Gutiérrez T, Rodríguez-Toscano E, Llorente C, et al. Neuropsychological, clinical and environmental predictors of severe mental disorders in offspring of patients with schizophrenia. *European Archives of Psychiatry and Clinical Neuroscience.* 2020;270(6):739-748.
224. Santucci AK, Singer LT, Wisniewski SR, et al. Impact of prenatal exposure to serotonin reuptake inhibitors or maternal major depressive disorder on infant developmental outcomes. *Journal of Clinical Psychiatry.* 2014;75(10):1088-1095.
225. Santucci AK, Singer LT, Wisniewski SR, et al. One-year developmental outcomes for infants of mothers with bipolar disorder. *Journal of Clinical Psychiatry.* 2017;78(8):1083-1090.
226. Saxena K, Simonetti A, Verrico CD, et al. Neurocognitive Correlates of Cerebellar Volumetric Alterations in Youth with Pediatric Bipolar Spectrum Disorders and Bipolar Offspring. *Curr Neuropharmacol.* 2023;21(6):1367-1378.
227. Schreiber H, Stolz-Born G, Heinrich H, et al. Attention, cognition, and motor perseveration in adolescents at genetic risk for schizophrenia and control subjects. *Psychiatry Research.* 1992;44(2):125-140.
228. Schreiber H, Stolz-Born G, Born J, et al. Visually-guided saccadic eye movements in adolescents at genetic risk for schizophrenia. *Schizophr Res.* 1997;25(2):97-109.
229. Schubert EW, McNeil TF. Neuropsychological impairment and its neurological correlates in adult offspring with heightened risk for schizophrenia and affective psychosis. *American Journal of Psychiatry.* 2005;162(4):758-766.
230. Segura AG, Serna Edl, Sugranyes G, et al. Polygenic risk scores mediating functioning outcomes through cognitive and clinical features in youth at family risk and controls. *European Neuropsychopharmacology.* 2024;81:28-37.
231. Sharma A, Camilleri N, Grunze H, et al. Neuropsychological study of IQ scores in offspring of parents with bipolar I disorder. *Cognitive Neuropsychiatry.* 2017;22(1):17-27.
232. Simonetti A, Kurian S, Saxena J, et al. Cognitive correlates of impulsive aggression in youth with pediatric bipolar disorder and bipolar offspring. *Journal of Affective Disorders.* 2021;287:387-396.
233. Singh MK, Leslie SM, Bhattacharjee K, et al. Vulnerabilities in sequencing and task switching in healthy youth offspring of parents with mood disorders. *Journal of Clinical & Experimental Neuropsychology: Official Journal of the International Neuropsychological Society.* 2018;40(6):606-618.
234. Spang KS, Ellersgaard D, Hemager N, et al. Executive functions in 7-year-old children of parents with schizophrenia or bipolar disorder compared with controls: The Danish High Risk and Resilience Study-VIA 7, a population-based cohort study. *European Child & Adolescent Psychiatry.* 2021;30(12):1871-1884.
235. Sunew EY. *Emotional intelligence in school-aged children: Relations to early maternal depression and cognitive functioning.* University of Washington; 2004.
236. Taylor L, Ingram RE. Cognitive reactivity and depressotypic information processing in children of depressed mothers. *J Abnorm Psychol.* 1999;108(2):202-210.
237. Topal Z, Demir N, Tufan E, et al. Emotional and cognitive conflict resolution and disruptive mood dysregulation disorder in adolescent offspring of parents diagnosed with major depressive disorder, bipolar disorder, and matched healthy controls. *Nordic Journal of Psychiatry.* 2021;75(6):427-436.

238. Traill SK. *Cognitive vulnerability to depression: Attention and memory biases in never-depressed daughters of depressed mothers.* Stanford University; 2002.
239. Veddum L, Greve AN, Andreassen AK, et al. Development of social responsiveness and theory of mind in children of parents with schizophrenia or bipolar disorder. *Schizophrenia Research: Cognition.* 2022;28:100242.
240. Venezia R. *Neurocognition in individuals at high familial risk for major depressive disorder*, St. John's University (New York); 2021.
241. Ver Loren van Themaat AH, Hemager N, Korsgaard Johnsen L, et al. Development of visual attention from age 7 to age 12 in children with familial high risk for schizophrenia or bipolar disorder. *Schizophrenia Research.* 2021;228:327-335.
242. Versace A, Ladouceur CD, Romero S, et al. Altered development of white matter in youth at high familial risk for bipolar disorder: a diffusion tensor imaging study. *J Am Acad Child Adolesc Psychiatry.* 2010;49(12):1249-1259, 1259.e1241.
243. Watt NF, Grubb TW, Erlenmeyer-Kimling L. Social, emotional, and intellectual behavior at school among children at high risk for schizophrenia. *Journal of Consulting and Clinical Psychology.* 1982;50(2):171-181.
244. Weissman MM, John K, Merikangas KR, et al. Depressed parents and their children. General health, social, and psychiatric problems. *American Journal of Diseases of Children.* 1986;140(8):801-805.
245. Welge JA, Saliba LJ, Strawn JR, et al. Neurofunctional Differences Among Youth With and at Varying Risk for Developing Mania. *J Am Acad Child Adolesc Psychiatry.* 2016;55(11):980-989.
246. Whiffen VE, Gotlib IH. Infants of postpartum depressed mothers: temperament and cognitive status. *J Abnorm Psychol.* 1989;98(3):274-279.
247. Whitney J, Howe M, Shoemaker V, et al. Socio-emotional processing and functioning of youth at high risk for bipolar disorder. *J Affect Disord.* 2013;148(1):112-117.
248. Winters KC, Stone AA, Weintraub S, et al. Cognitive and attentional deficits in children vulnerable to psychopathology. *J Abnorm Child Psychol.* 1981;9(4):435-453.
249. Wolf LE, Cornblatt BA, Roberts SA, et al. Wisconsin Card Sorting deficits in the offspring of schizophrenics in the New York High-Risk Project. *Schizophrenia Research.* 2002;57(2):173-182.
250. Woody ML, Burkhouse KL, Gibb BE. Overgeneral autobiographical memory in children of depressed mothers. *Cognition and Emotion.* 2015;29(1):130-137.
251. Worland J, Hesselbrock V. The intelligence of children and their parents with schizophrenia and affective illness. *Child Psychology & Psychiatry & Allied Disciplines.* 1980.
252. Yazkan-Akgul G, Perdahli-Fis N. Are neurocognitive abilities and social cognition related to social and role functioning in familial high risk group for psychosis? *Early intervention in psychiatry.* 2022;16(12):1335-1344.
253. Yoshida K, Marks MN, Craggs M, et al. Sensorimotor and cognitive development of infants of mothers with schizophrenia. *British Journal of Psychiatry.* 1999;175(OCT.):380-387.
254. Zhou Y, Liu J, Driesen N, et al. White Matter Integrity in Genetic High-Risk Individuals and First-Episode Schizophrenia Patients: Similarities and Disassociations. *BioMed Research International.* 2017;2017(1):3107845.
255. Zhu T, Simonetti A, Ouyang M, et al. Disrupted white matter microstructure correlates with impulsivity in children and adolescents with bipolar disorder. *Journal of Psychiatric Research.* 2023;158:71-80.
